# Dual gene‐activated dermal scaffolds regulate angiogenesis and wound healing by mediating the coexpression of VEGF and angiopoietin‐1

**DOI:** 10.1002/btm2.10562

**Published:** 2023-06-25

**Authors:** Tingting Weng, Min Yang, Wei Zhang, Ronghua Jin, Sizhan Xia, Manjia Zhang, Pan Wu, Xiaojie He, Chunmao Han, Xiong Zhao, Xingang Wang

**Affiliations:** ^1^ Department of Burns & Wound Care Centre Second Affiliated Hospital of Zhejiang University School of Medicine Hangzhou China; ^2^ The Key Laboratory of Severe Trauma and Burns of Zhejiang Province Hangzhou China; ^3^ Department of Burn and Plastic Surgery Children's Hospital, Zhejiang University School of Medicine, National Clinical Research Center for Child Health, National Children's Regional Medical Center Hangzhou China; ^4^ The First Clinical Medical College, Zhejiang Chinese Medical University Hangzhou China

**Keywords:** angiogenesis, angiopoietin‐1, dual gene‐activated scaffolds, VEGF, wound healing

## Abstract

The vascularization of dermal substitutes is a key challenge in efforts to heal deep skin defects. In this study, dual gene‐activated dermal scaffolds (DGADSs‐1) were fabricated by loading nanocomposite particles of polyethylenimine (PEI)/multiple plasmid DNAs (pDNAs) encoding vascular endothelial growth factor and angiopoietin‐1 at a ratio of 1:1. In a similar manner, DGADSs‐2 were loaded with a chimeric plasmid encoding both VEGF and Ang‐1. In vitro studies showed that both types of DGADSs released PEI/pDNA nanoparticles in a sustained manner; they demonstrated effective transfection ability, leading to upregulated expression of VEGF and Ang‐1. Furthermore, both types of DGADSs promoted fibroblast proliferation and blood vessel formation, although DGADSs‐1 showed a more obvious promotion effect. A rat full‐thickness skin defect model showed that split‐thickness skin transplanted using a one‐step method could achieve full survival at the 12th day after surgery in both DGADSs‐1 and DGADSs‐2 groups, and the vascularization time of dermal substitutes was significantly shortened. Compared with the other three groups of scaffolds, the DGADSs‐1 group had significantly greater cell infiltration, collagen deposition, neovascularization, and vascular maturation, all of which promoted wound healing. Thus, compared with single‐gene‐activated dermal scaffolds, DGADSs show greater potential for enhancing angiogenesis. DGADSs with different loading modes also exhibited differences in terms of angiogenesis; the effect of loading two genes (DGADSs‐1) was better than the effect of loading a chimeric gene (DGADSs‐2). In summary, DGADSs, which continuously upregulate VEGF and Ang‐1 expression, offer a new functional tissue‐engineered dermal substitute with the ability to activate vascularization.

## 1 INTRODUCTION

Skin defects triggered by various acute and chronic factors (e.g., trauma, mechanical injuries, burns, chronic ulcers, and tumor resection) are prevalent in clinical practice^1^; they greatly affect public health, as well as economic and social development. A dermal substitute is a template for dermal regeneration that plays a crucial role in dermal reconstruction. Its advent provides an ideal method for the treatment of deep skin defects. Nevertheless, delayed or insufficient vascularization of dermal substitutes remains a key challenge.^2^ This limitation primarily manifests as the slow growth of new blood vessels during the early stage of dermal substitute transplantation. There is an urgent need to accelerate the vascularization of dermal substitutes; this will enhance repair efficiency.

The vascularization process for a dermal substitute implanted into a wound is similar to the vascularization that occurs during wound healing. This complex process involves interactions among various cells (e.g., fibroblasts, endothelial cells, and pericytes) and various bioactive factors (e.g., vascular endothelial growth factor [VEGF], fibroblast growth factor, epidermal growth factor [EGF], platelet‐derived growth factor [PDGF], angiopoietin‐1 [Ang‐1], extracellular matrix, and proteolytic enzymes).^3^ To ensure the rapid vascularization of dermal substitutes, various methods have been established to promote vascularization; such methods include optimization of the scaffold structure,^4^ pre‐seeding of vascular endothelial cells or stem cells,^5^ and introduction of angiogenic factors.^6^ The most powerful approach involves the incorporation of angiogenic factors such as VEGF, PDGF, and basic fibroblast growth factor (bFGF). However, the direct application of these expensive angiogenic factors often leads to unsatisfactory effects, primarily because such factors are unstable, short‐lived, and susceptible to inactivation; thus, they cannot continuously promote vascularization.^7^ DNA is much more stable than these angiogenic factors; a scaffold that can release DNA encoding angiogenic factors provides a powerful alternative to the direct delivery of bioactive proteins. In recent years, gene‐activated scaffolds (GASs) have been increasingly used in tissue engineering.^8^, ^9^ GASs directly introduce exogenous genes into the body without in vitro cell culture; they use gene vectors for in vivo transfection to achieve the sustained local expression of bioactive factors necessary to regulate angiogenesis and wound healing.^10^, ^11^ Guo et al. developed a gene‐activated bilayer dermal equivalent called *N*,*N*,*N*‐trimethyl chitosan chloride (TMC; a cationic gene delivery vector) to form complexes with the plasmid DNA encoding VEGF‐165; these complexes were then incorporated into a collagen–chitosan/silicone membrane scaffold. In their study, the TMC/pDNA‐VEGF group had the highest levels of VEGF expression (both mRNA and protein), which yielded the highest densities of newly formed and mature vessels.^10^ Wang et al. reported the fabrication and application of dextran, a sophisticated hydrogel made from chemically modified hyaluronic acid, along with β‐cyclodextrin‐integrating resveratrol (Res) and VEGF plasmid (pVEGF) as the anti‐inflammatory and proangiogenic components for burn wounds. Gel‐Res/pDNA‐VEGF‐treated wounds demonstrated accelerated wound healing, characterized by decreased inflammation and increased angiogenesis, compared with untreated wounds and gel‐alone‐treated wounds over a 21‐day period.^12^ In addition, Lou et al. constructed gene‐activated bilayer dermal equivalents, which have good biocompatibility. Gene‐activated bilayer dermal equivalents were fabricated by loading nano‐sized complexes of Lipofectamine 2000/plasmid DNA encoding VEGF into a collagen–chitosan scaffold/silicone membrane bilayer dermal equivalent. The gene‐activated bilayer dermal equivalents demonstrated versatile potential to promote sustained healing of diabetic chronic wounds by upregulating the expression of VEGF.^9^

The previous studies were mainly focused on the application of pVEGF. VEGF is a key regulator of neovascularization; it has an important role in the vascular formation process. VEGF can specifically bind to the VEGFR‐2 receptor on the surface of endothelial cells to promote endothelial cell migration and proliferation, followed by primary lumen formation. Nevertheless, these new blood vessels exhibit immature structure and function, characterized by a narrow lumen, high vessel wall permeability, and tendency to leak easily.^13^, ^14^ Thus, vascular maturation is necessary to improve the function of new blood vessels. Pericytes play a vital role in vascular maturation. Pericytes stabilize the newly formed vascular structure by direct or paracrine interactions with endothelial cells during a late stage of vascularization, thus promoting new blood vessel maturation.^15^ There is increasing evidence that the proliferation, migration, and transformation of pericytes are regulated by numerous signaling pathways, such as the Ang‐1/Tie‐2,^16^, ^17^ PDGF‐B/PDGFRβ,^18^ and transforming growth factor beta pathways. Among these pathways, Ang‐1 and its receptor Tie‐2 have a critical role in vascular maturation and remodeling. Ang‐1 has been shown to recruit pericytes and smooth muscle cells, stimulate pericytes to wrap around endothelial cells, increase basement membrane deposition, facilitate mature vessel formation, and reduce plasma protein leakage.^13^ Ang‐1 initiates positive regulation of vascular differentiation and maturation through specific binding to Tie‐2 receptors; its pro‐vascularization effect also depends on the presence of VEGF.^13^, ^19^ In other words, Ang‐1 was unable to play a role in promoting vascularization without VEGF. The coexpression of VEGF and Ang‐1 in the skin of transgenic mice reportedly generated larger, leakage‐resistant vessels.^20^ Thus, VEGF and Ang‐1 have important synergistic effects during angiogenesis and maturation, and this synergy exists in a chronological order. By using the synergy between these two factors, along with transgene therapy to construct a dual‐gene active dermal scaffold, precise regulation of vascular formation and maturation can be realized.

Our group previously developed a reinforced dermal regeneration template (PLGAm/CCSs). PLGAm/CCSs have a suitable three‐dimensional (3D) porous structure, biological properties similar to the properties of skin tissue, and the ability to rapidly induce tissue regeneration; they thus offer an ideal material for constructing dermal substitutes.^21^, ^22^ To achieve efficient vascularization of the dermal substitute, we loaded PEI/pDNA nanoparticles (NPs) onto PLGAm/CCSs and developed two scaffolds that contained dual gene‐activated VEGF and Ang‐1. Subsequently, we performed physicochemical characterization and cellular experiments on each group of scaffolds. We evaluated the optimal gene loading, and early angiogenesis of DGADSs via subcutaneous implantation in Sprague–Dawley rats. Finally, a full‐thickness skin defect model in Sprague–Dawley rats was used to evaluate the effects of DGADSs on angiogenesis, vascular maturation, and wound healing during the vascularization of scaffolds; the underlying mechanisms were also explored.

## 2 EXPERIMENTAL

### 2.1 Materials

PLGA‐knitted mesh and Type I collagen were provided by Zhende Medical Devices Co., Ltd. (Shaoxing, China), and chitosan (deacetylation degree ≥85%) was purchased from Sigma‐Aldrich (St. Louis, MO). Plasmid encoding VEGF‐165 (pVEGF), plasmid encoding angiopoietin‐1 (pAng‐1), chimeric plasmid encoding both VEGF‐165 and angiopoietin‐1 (pVEGF/Ang‐1), and blank plasmid encoding GFP (pEGFP) were prepared and tested by Hangzhou Niubei Biotechnology Co., Ltd. (Hangzhou, China). The plasmids were amplified and purified in *Escherichia coli*, using a Qiagen Endofree Plasmid Maxi kit (Qiagen, ID12362, Germany), and stored at −20°C before use. PEI was purchased from Hangzhou Aiting Biotechnology Co., Ltd. (Hangzhou, China).

### 2.2 Cell culture

Fbs cells were obtained from discarded human foreskin.^23^ Briefly, human foreskin was sterilized in povidone iodine for several minutes, then submerged in a 0.25% dispase enzyme solution overnight at 4°C. After the foreskin had been washed twice with phosphate‐buffered saline (PBS), the epidermal layer of the human foreskin was removed and the dermis was cut into small pieces. The small pieces were then transferred into a 0.25% collagenase I enzyme solution at 37°C for 2 h, after which 10% fetal bovine serum (FBS; Gibco, USA) was added to the solution to stop digestion; this solution was filtered to obtain a cell suspension. The floating cells were centrifuged at 1000 rpm for 10 min, then cultured in Dulbecco's modified Eagle's medium (DMEM; Sigma‐Aldrich) supplemented with 10% FBS and 1% penicillin/streptomycin at 37°C with 5% CO_2_ in a cell culture incubator.

HUVECs were purchased from the American Type Culture Collection (Nashua, NH). They were also cultured in DMEM containing 10% FBS and 1% penicillin/streptomycin at 37°C with 5% CO_2_.

### 2.3 Preparation of PEI/pDNA NPs

A high‐concentration plasmid DNA solution was diluted with Opti‐MEM™ (Sigma‐Aldrich) to a concentration of 1000 ng/μL to prepare the DNA premix. PEI reagent was then added using a volume ratio of 1:3 for thorough mixing. The mixture was incubated for 15 min at room temperature to form stabilized PEI/pDNA NPs. The PEI/pDNA NPs solution was dropped on a 200‐mesh copper mesh, dried, and observed using a transmission electron microscopy (TEM; Hitachi, Japan).

### 2.4 Fabrication of GASs

PLGAm/CCSs were prepared in accordance with previously described procedures.^21^ Briefly, collagen and chitosan at a mass ratio of 9:1 were dissolved in 0.5 M acetic acid solution to generate a mixture with a total concentration of 0.5% (w/v). The PLGA mesh was cut into designated sizes and extended on a flat plate (custom model). The collagen–chitosan solution was carefully poured, and the mesh remained unfolded. The composite was stored at −4°C for 24 h, frozen at −25°C for 3 h, and then lyophilized for 16 h to obtain the PLGAm/CCSs. The PLGAm/CCSs were pre‐treated via 1‐ethyl‐3‐(3‐dimethyl aminopropyl) carbodiimide/*N*‐hydroxysuccinimide (Yuanye Biotechnology Co., Ltd., Shanghai, China) cross‐linking,^24^ then lyophilized for future use. Subsequently, 100‐μL suspensions of PEI/pDNA NPs (as discussed in Section 2.2) were injected into PLGAm/CCSs via physical adsorption and a multipoint injection method, followed by overnight incubation at 4°C to facilitate the adsorption of nanoparticles. Finally, GASs were obtained. Four types of GASs were prepared in this study: PEI/pVEGF and PEI/pAng‐1 NPs loaded 1:1 with PLGAm/CCSs (DGADSs‐1); PEI/pVEGF/Ang‐1 (chimeric plasmid) NPs loaded with PLGAm/CCSs (DGADSs‐2); and two control groups comprising PLGAm/CCSs loaded with PEI/pVEGF NPs and PEI/pDNA‐EGFP NPs, respectively.

After lyophilization for 24 h, GASs were cut into 1 mm × 3 mm × 1 mm samples with a sharp blade, dried and gold‐sprayed, and then placed under a scanning electron microscope (Hitachi, Japan) to observe the distribution of DNA‐PEI NPs and the pore size of the scaffold.

### 2.5 Release of PEI/pDNA NPs from GASs

A release assay was carried out to determine the release kinetics of pDNA from PLGAm/CCSs. GASs were immersed in 2 mL of sterile PBS at 37°C. At the scheduled time points, 200 μL of supernatant was collected for analysis, and the same volume of fresh PBS was replenished. Then, a 100 μL aliquot was used to determine the amount of pDNA in the sustained‐release solution and a 100 μL aliquot was used to evaluate in vitro transfection of the PEI/pDNA NPs. An ultra‐trace nucleic acid protein detector (Jiapeng Technology Co., Ltd., Shanghai, China) was used to detect the amount of pDNA in the sustained‐release solution and draw the cumulative release curve of PEI/pDNA NPs.

### 2.6 Transfection efficiency in vitro

HUVECs were inoculated into a 24‐well plate at 10,000 cells/well and cultured for 24 h. The sustained‐release PEI/pDNA NPs were added at a ratio of 1 μg DNA/10,000 cells. At a predetermined time point, the culture was observed with a fluorescence microscope and digested with 0.25% Trypsin to obtain a single cell suspension. The transfection efficiency was detected by flow cytometry (BD Bioscience, Franklin Lakes, NJ). Non‐transfected cells were used as a negative control.

### 2.7 Cell proliferation assay

DGADSs‐1, DGADSs‐2, PEI/pVEGF, PEI/pEGFP, and a blank scaffold (no gene loading) were added to a 96‐well plate and immersed in culture medium for 12 h. Fbs cells in the logarithmic phase were collected and prepared as a cell suspension. The cell density was adjusted to 1 × 10^5^ cells/mL; the suspension was then added to 96‐well plates at 100 μL per well, with six parallel samples for each group at each time point. Subsequently, the samples were incubated at 37°C with 5% CO_2_, and the medium was changed at 2‐day intervals. After 1, 3, and 5 days, the proliferation of Fbs cells was tested using a Cell Counting Kit‐8 assay (Boster, Wuhan, China). Briefly, CCK‐8 solution and serum‐free DMEM were mixed in a volume ratio of 1:10 to obtain a working solution. After cells had been washed with PBS, they were submerged in the working solution and cultured at 37°C for 3 h. Finally, 100 μL of the CCK‐8 working solution were added to a 96‐well plate to measure the absorbance at 450 nm with a microplate reader (BioTek, Winooski, VT).

After 5 days of culture, EdU cell proliferation assays were performed (BeyoClick™ EdU Cell Proliferation Kit with Alexa Fluor 488, Beyotime, China). Briefly, five groups of scaffold‐cultured cells were incubated with EdU for 2 h, fixed with 4% paraformaldehyde for 15 min, and permeated with 0.3% Triton X‐100 for 15 min. The cells were incubated with the Click Reaction Mixture for 30 min at room temperature in the dark, then incubated with Hoechst 33342 for 10 min.

### 2.8 Tube formation assay

Matrigel (BD Bioscience) was melted at 4°C, centrifuged for several minutes, and gently mixed with a precooled pipette tip. A 96‐well plate was precooled in advance, and 50 μL of Matrigel was added to each well. When the HUVECs with different treatments in each group grew to 70%–80% confluency, they were trypsinized and resuspended in DMEM containing 10% FBS. Next, 50 μL of the resuspension were added to the Matrigel at a concentration of 40,000 cells/well in six wells. The samples were incubated at 37°C and blood vessel formation was observed at predetermined time points.

### 2.9 VEGF and Ang‐1 expression in vitro

VEGF and Ang‐1 expression levels in the HUVECs cultured in vitro in the four GAS groups were examined using enzyme‐linked immunosorbent assays (ELISAs). At the scheduled time points, the scaffolds were washed three times with PBS (pH 7.4), then homogenized in lysis buffer (0.1 M Tris–HCl, 2 mM ethylenediaminetetraacetic acid, and 0.1% Triton X‐100). Lysates were then centrifuged at 12,000 rpm for 5 min at 4°C to collect supernatants. The amounts of VEGF and Ang‐1 were analyzed using a VEGF ELISA Kit (Abcam, Cambridge, UK) and an Ang‐1 ELISA Kit (Abcam), in accordance with the manufacturer's instructions.

### 2.10 Angiogenesis‐related protein expression in vitro

DGADSs‐1, DGADSs‐2, and the blank scaffold (no gene loading) were sterilized, added to a 12‐well plate, and immersed in culture medium for 12 h. The HUVECs suspension was then added to 12‐well plates at 10,000 cells/well, and incubated at 37°C with 5% CO_2_. On day 5, the HUVEC proteins cultured on scaffolds in each group were collected for an angiogenesis antibody array‐membrane assay (43 targets, ab193655, Abcam).

### 2.11 Subcutaneous implantation model in rats

The experimental groups were as follows: DGADSs‐1, DGADSs‐2, PEI/pVEGF, and PEI/pEGFP. GASs with 0, 5, 10, and 15 μg of gene loading were prepared in each group and implanted subcutaneously in Sprague–Dawley rats for 21 days to identify the best gene loading method for scaffolds. The rats were first anesthetized by intraperitoneal injection of 2% pentobarbital sodium saline solution at 50 mg/kg. The hair in the operative area of each rat's dorsum was shaved with electric clippers, depilated with hair removal cream, and finally disinfected with 2.5% povidone iodine solution and 75% ethanol. Four back incisions (1 cm from the dorsal midline) were opened and unfolded; the four GAS groups underwent implantation separately. All implants were ≥2.0 cm from the adjoining wound. Blood flow signals of the implanted scaffolds were detected by a small animal ultrasonic Doppler flow meter (INDUS, Flint, MI) and the macroscopic appearance of the implanted scaffolds was recorded by a camera (Spectral Instruments Imaging, Tucson, AZ) on days 10 and 21 post‐implantation. Subsequently, samples from each group were harvested and maintained in a 4% paraformaldehyde solution and liquid nitrogen for further analysis.

### 2.12 Full‐thickness skin defect model in rats

The four groups of scaffolds (DGADSs‐1, DGADSs‐2, PEI/pVEGF, and PEI/pEGFP with gene loadings of 10 μg each) were sterilized for subsequent use. The rats were numbered one by one and then grouped using a random number table. The rats were anesthetized by intraperitoneal injection of 2% pentobarbital sodium saline solution at 50 mg/kg and the hair in the operative area was prepared as above. Two circular full‐thickness skin defect wounds (1.2 cm in diameter) were made bilaterally at 1 cm from the dorsal midline. The sterile scaffolds were successively transplanted to the corresponding wounds; the autogenous split‐thickness skin (STS) was then trimmed and sutured to the skin defect. The wound was bandaged, and the dressing was changed within 24 h. The transplantation sites were photographed and harvested on the specified day; the samples were stored in a 4% paraformaldehyde solution, a 2.5% glutaraldehyde solution, and liquid nitrogen, respectively, for follow‐up histological analyses and molecular biological detection. Rats procedures were performed in accordance with protocols approved by the Animal Care and Use Committee of the Second Affiliated Hospital, College of Medicine, Zhejiang University (Animal protocol number: 2017‐261).

### 2.13 TEM observation

The samples were fixed overnight in 2.5% glutaraldehyde, then washed with PBS. Subsequently, the samples were fixed in 1% osmic acid solution (1 h) and washed with PBS, then subjected to ethanol gradient dehydration, infiltration, embedding, and slicing. The sections were stained with lead citrate and uranyl acetate solutions for 5 min, then observed via TEM (Hitachi H‐7650).

### 2.14 Histological analysis

The samples were fixed in 4% paraformaldehyde solution, embedded in paraffin, and sectioned. Hematoxylin and eosin (H&E) staining, Masson's trichrome staining, and Sirius Red staining were performed in accordance with the manufacturer's standardized protocols.

### 2.15 Immunofluorescence

Histological sections were deparaffinized, washed with PBS (three times for 5 min each), and blocked with 5% bovine serum albumin for 30 min. Then, the sections were incubated with anti‐CD31 (1:200, ab222783, Abcam), anti‐α‐SMA (1:200, ab7817, Abcam) and anti‐VEGFR‐2 (1:200, ab2349, Abcam) primary antibodies at 4°C overnight. After sections had been washed three times with PBS, they were incubated for 2 h at room temperature with secondary tetramethylrhodamine‐conjugated goat anti‐mouse antibody (1:300, Invitrogen, Waltham, MA) and fluorescein isothiocyanate‐conjugated goat anti‐rabbit antibody (1:300, Invitrogen). Images were captured using a DM2500 microscope equipped with a Leica DFC490 camera (Leica, Wetzlar, Germany).

### 2.16 Angiogenesis‐related protein expression in vivo

An Angiogenesis Antibody Array‐Membrane (ab193655, Abcam) was used to detect the expression of angiogenesis‐related factors in the wound tissue in the four GAS groups at 2 weeks post‐transplantation.

### 2.17 Statistical analysis

Values are expressed as means ± standard deviations. Statistical analyses were performed using two‐way *t*‐tests between two groups or by one‐way analysis of variance among multiple groups. Significance levels were set at *P* < 0.05 (*) and *P* < 0.01 (**).

## 3 RESULTS

### 3.1 Macroscopic shape and microstructure of GASs

**FIGURE 1:**
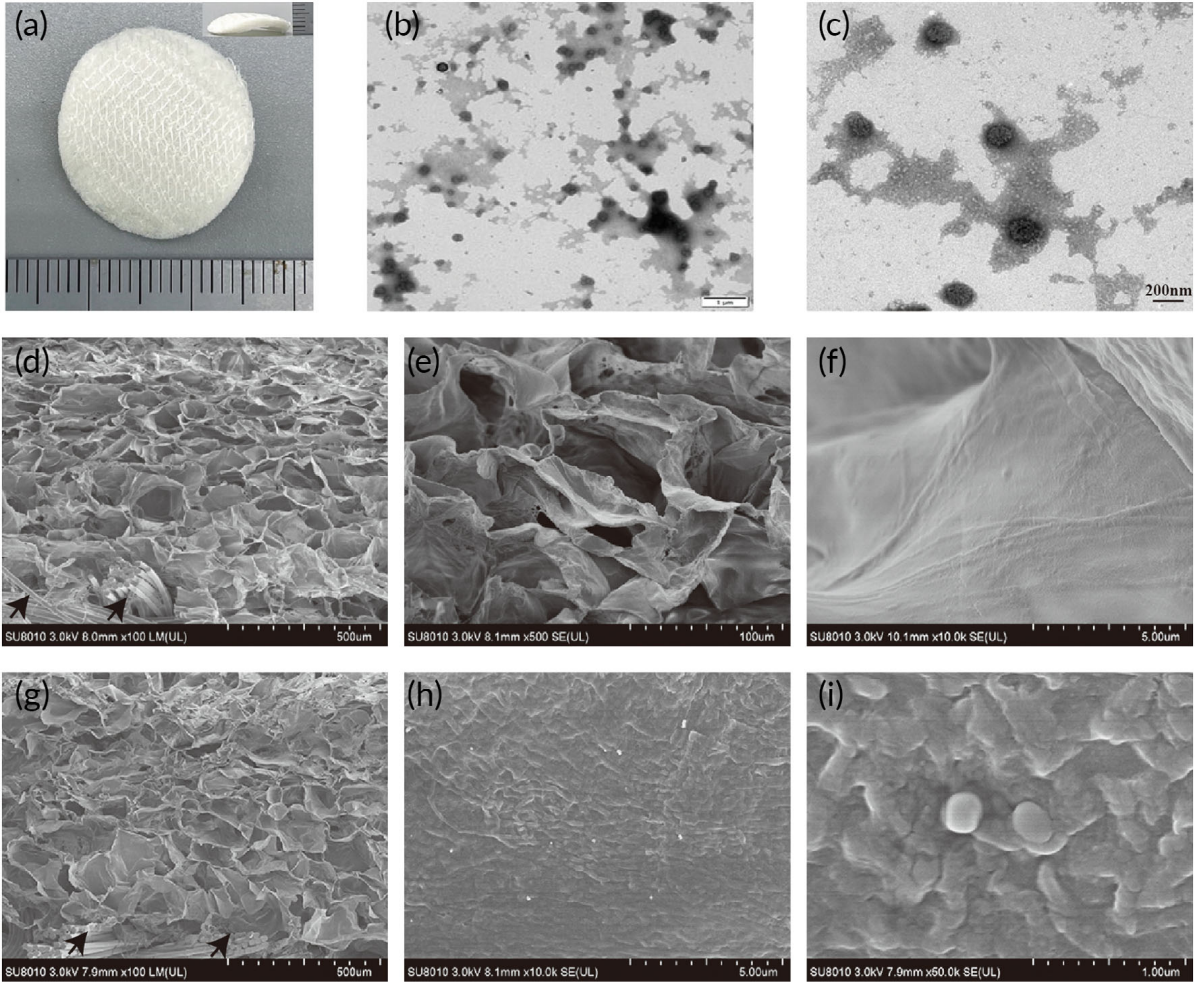
Macroscopic shape and microstructure of GASs. (a) Gross view of GASs, Scale bar = 2 mm. (b,c) are TEM images of PEI/pDNA‐complexes with different magnifications, the scale bars are 1 μm and 200 nm, respectively. (d–f) are the SEM images of blank scaffolds with different magnifications. (g–i) are the SEM images of PEI/pDNA nanoparticles‐loaded PLGAm/CCS with different magnifications.

The general appearance of the GASs is shown in Figure 1a. The overall appearance of the scaffold was white and generally compact after secondary freeze‐drying, with a thickness of 2 mm. TEM revealed that the PEI/pDNA complexes had polymerized into nanospheres, with a mean diameter of 145 ± 14.1 nm (Figure 1b,c). Nanoparticles can better promote the introduction of exogenous genes into cells for nuclear translocation.^25^ Scanning electron microscope observations of the cut surfaces of GASs revealed that the scaffolds had an obvious double‐layer structure, with a bottom layer of PLGA‐knitted mesh (black arrow). The upper layer was thick; it comprised a CCS porous structure with a mean pore size of ~131 ± 32 μm, and a generally uniform pore size suitable for cell infiltration and nutrient exchange (Figure 1d,e). High‐power microscopy showed that the CCS pore walls of the blank scaffolds were smooth in appearance, and no particles were attached (Figure 1f). After incorporation of PEI/pDNA nanoparticles, the GASs exhibited a similar porous structure (Figure 1g), with a mean pore size of 127 ± 20 μm; this was slightly smaller than the mean pore size of the blank scaffold because of further freezing and lyophilization after PEI/pDNA NPs loading. Magnified scanning electron microscope images showed that many PEI/pDNA NPs adhered to the pore walls of the CCSs (Figure 1h,i), with a generally uniform distribution.

### 3.2 In vitro release of PEI/pDNA NPs

**FIGURE 2:**
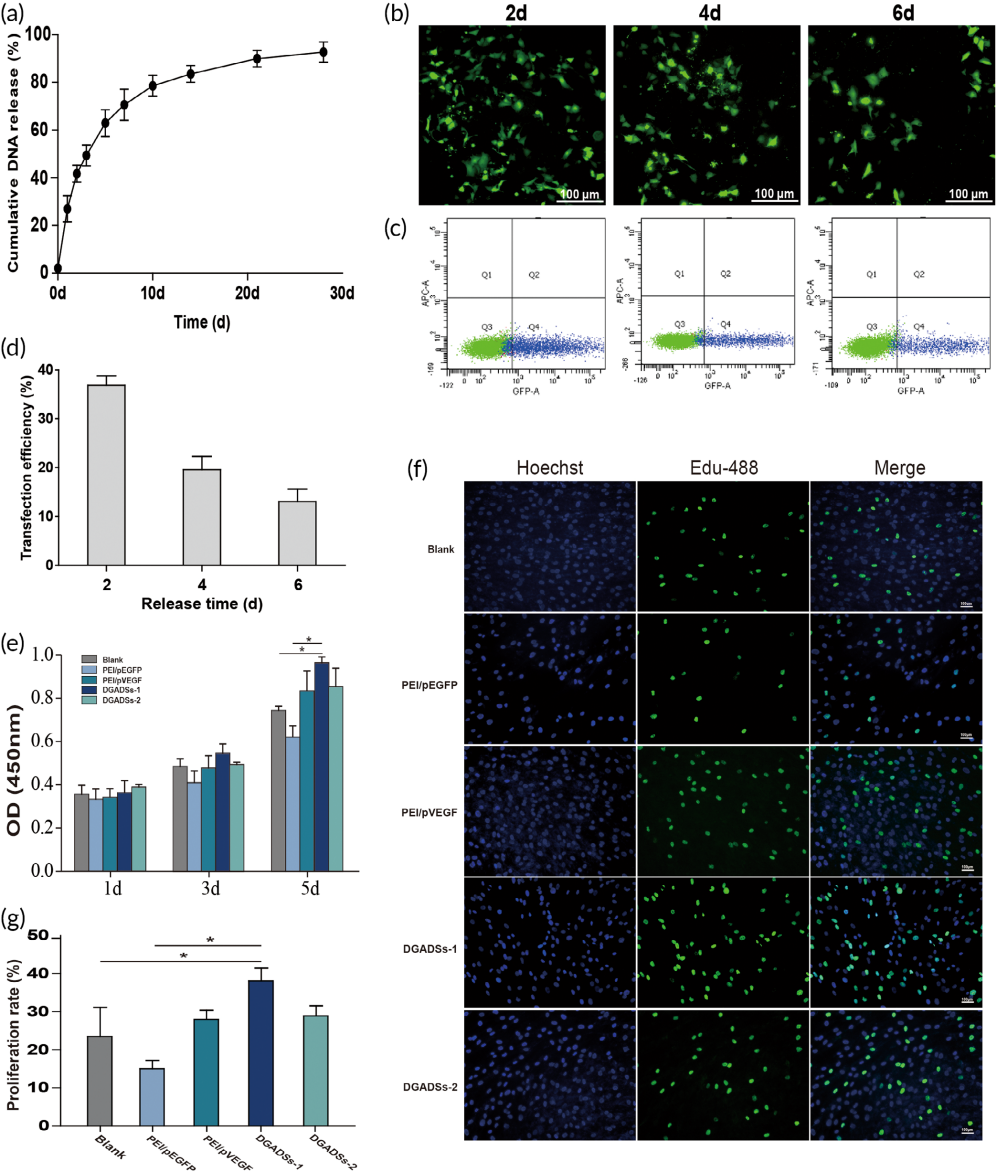
Release pattern, transfection efficiency, and cell proliferation (a) Cumulative release of PEI/pDNA nanoparticles from GASs. The part figure (b) are the fluorescence images, showing the HUVECs transfected by the PEI/pDNA nanoparticles released from GASs at 2, 4, and 6 day, Scale bar = 100 μm. (c) The flow images, showing the HUVECs transfected by the PEI/pDNA nanoparticles released from GASs at 2, 4, and 6 day. (d) The quantification of transfection efficiency at 2, 4, and 6 day. (e) The cell proliferation of Fbs in 3D static culture with five groups scaffolds by CCK‐8 on days 1, 3, and 5, *P < 0.05. (f,g) The EdU staining and quantitative analysis of five groups scaffolds on day 5, *P < 0.05, Scale bar = 100 μm.

Figure 2a shows the cumulative release of PEI/pDNA NPs from GASs. Overall, the curve showed a linear release over time for the first 7 days, with a high release rate. By day 7, 70% of the nanoparticles were released because of an imbalance in DNA concentrations between the scaffolds and solutions.^26^ The release rate slowed after 7 days, and the cumulative release of PEI/pDNA NPs reached 90% at 28 days. These results imply that the GASs prepared by physical adsorption in this study had sustained‐release properties, whereby the sustained release of PEI/pDNA NPs could persist for >28 days.

### 3.3 In vitro transfection efficiency

The sustained‐release PEI/pDNA NPs were transfected into HUVECs to evaluate their transfection efficiency in vitro (Figure 2b,c). The number of GFP‐positive HUVECs gradually decreased with increasing release time. On day 2, the transfection efficiency of the PEI/pDNA NPs reached its maximum, with a transfection efficiency of 36.8%; it then gradually decreased. This was presumably because large amounts of PEI/pDNA NPs released from GASs remained structurally intact for a short period and the pDNA maintained its transfection capacity, resulting in a continuous increase in transfection efficiency. However, with increasing culture time, the transfection efficiency of PEI/pDNA NP decreased. This finding might be due to the long‐term deposition of PEI/pDNA NP in medium, which lead to the structural change of PEI/pDNA NP, thereby decreased their ability to penetrate cells.^8^ On days 4 and 6, the in vitro transfection efficiencies of the sustained‐release PEI/pDNA NPs were 19.2% and 13.1%, respectively; these findings indicated achievement of the expected transfection efficiency level (Figure 2d).

### 3.4 Cell proliferation

As one of the main cell types involved in wound healing, Fbs provide a reliable means of evaluating the biocompatibility of GASs because such cells are ubiquitous at various stages of healing. CCK‐8 was used to compare cell proliferation among the five groups of scaffolds in 3D statically cultured Fbs on days 1, 3, and 5. Figure 2e shows that the number of viable cells of all scaffold groups increased with increasing time in the 3D culture. On days 1 and 3, no significant difference was observed among the five groups. The survival rates of the DGADSs‐1, DGADSs‐2, and PEI/pVEGF groups were significantly higher than the survival rates of the PEI/pEGFP and blank scaffold groups on day 5; the difference in the DGADSs‐1 group was statistically significant (*P* < 0.05). Figure 2f,g show EdU staining and quantification on day 5. The results indicate that DGADSs loaded with VEGF and Ang‐1 plasmids can promote the proliferation of Fbs; the effect in the DGADSs‐1 group was statistically significant (*P* < 0.05), consistent with the CCK‐8 staining results. Thus, all five scaffold groups had good biocompatibility. Among them, DGADSs‐1, DGADSs‐2, and PEI/pVEGF loaded with functional genes pVEGF or pAng‐1 more effectively promoted the proliferation of Fbs; the DGADSs‐1 group showed the greatest effect. The PEI/pEGFP group had the lowest cell activity, which may have been related to the cytotoxicity of PEI/pEGFP particles. In general, cationic polymers (e.g., polyethyleneimine and trimesoyl chloride) are cytotoxic. Such polymers may interact with negatively charged glycoproteins on the cell membrane, thus interfering with cell membrane structure and resulting in cytotoxicity.^27^

### 3.5 Tube formation assay

**FIGURE 3:**
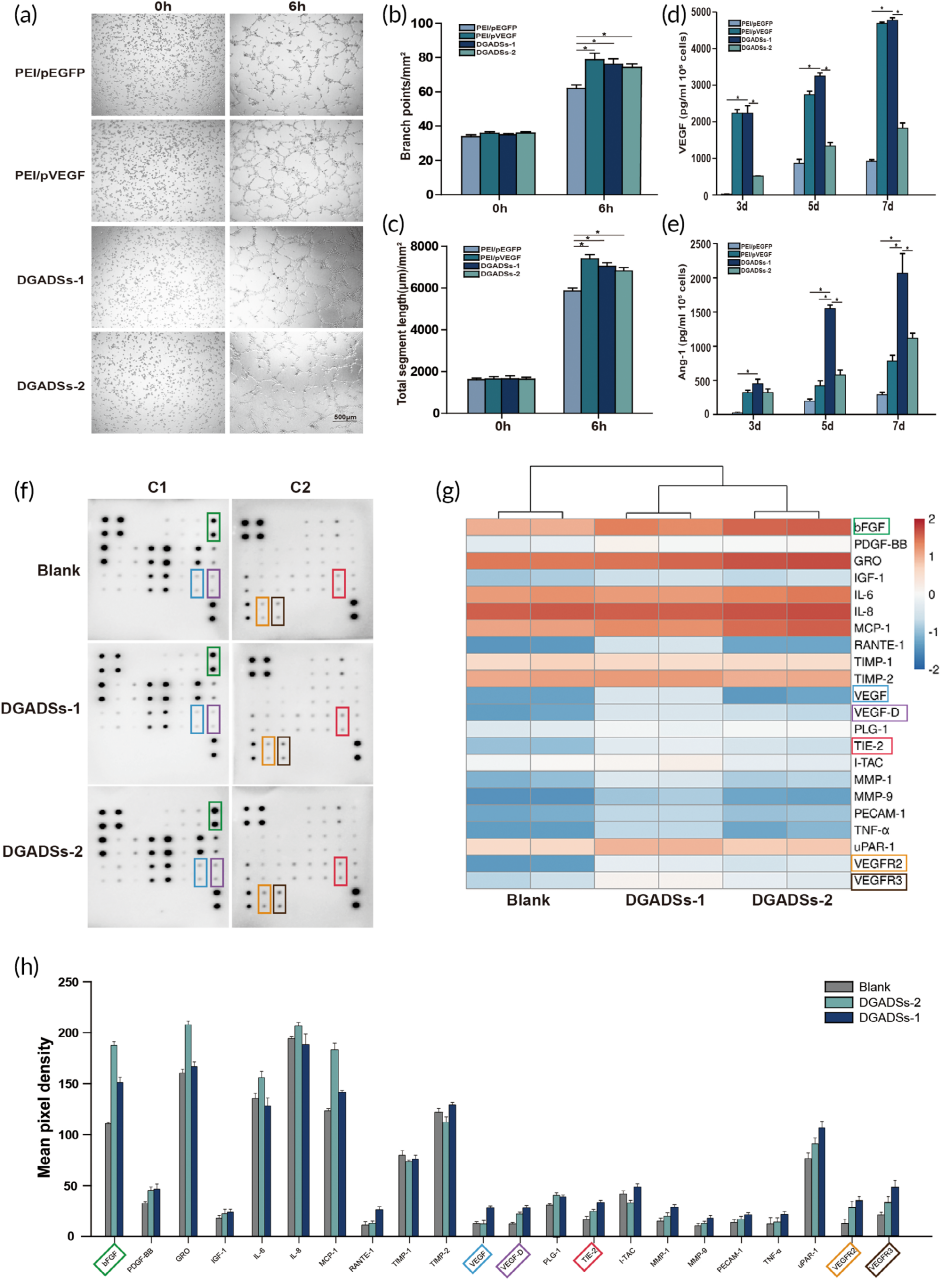
Tube‐formation assay and angiogenesis‐related protein expression in vitro. (a) Representative images of 4 groups of GASs treated HUVECs in tube formation assay. (b) The number of branch points of HUVEC treated with four groups of GASs, *n* = 6. (c) The total segment length of HUVEC treated with four groups of GASs. *P < 0.05, Scale bar = 500 μm, *n* = 6. (d) In vitro VEGF expression of HUVECs after being cultured in different GASs for 3, 5, and 7 days. *P < 0.05, *n* = 6. (e) In vitro Ang‐1 expression of HUVECs after being cultured in different GASs for 3, 5, and 7 days. *P < 0.05, *n* = 6. (f) Proteome array blot of the expression of angiogenesis‐related protein in HUVECs cultured with DGADSs after 5 days at initial analysis. Spot data on C1 and C2 membranes from arrays of angiogenic antibodies in blank groups, DGADSs‐1, and DGADSs‐2 (Top‐to‐bottom). C1 and C2 membranes are antibody array chips containing different human angiogenesis proteins respectively (Detailed results in Figure S7). (g) Heatmap recapitulating the differentially expressed in angiogenic protein between DGADSs‐1, DGADSs‐2, and Blank groups. Red represents relatively high expression, and blue represents relatively low expression. Proteins of interest are highlighted in colored outlines. (h) The relative performance in angiogenic protein expression between three groups of GASs was calculated.

HUVECs retain the ability to divide and rapidly migrate; they can respond to angiogenic signals. Here, we used a tube formation assay to study the ability of GASs to promote angiogenesis in vitro. The total segment length and the number of branch points of HUVECs were greater in the groups treated with DGADSs‐1, DGADSs‐2, and PEI/pVEGF than in the group treated with PEI/pEGFP, at both 3 and 6 h (*P* < 0.05) (Figure 3a,c). Overall, the results indicate that DGADSs‐1, DGADSs‐2, and PEI/pVEGF promote blood vessel formation by HUVECs.

### 3.6 VEGF and Ang‐1 expression in vitro

Because they are natural VEGF and Ang‐1 receptor cells,^28^, ^29^ HUVECs were chosen to assess the effect of GASs on VEGF and Ang‐1 expression. Figure 3d,e show that the expression levels of VEGF and Ang‐1 in cells increased over time in four scaffold groups, consistent with the cell proliferation results described in a previous section. Notably, the expression levels of VEGF and Ang‐1 were significantly higher in the DGADSs‐1 group than in the other three groups at each time point (*P* < 0.05); this presumably resulted from the in vitro transfection of VEGF and Ang‐1 plasmids. During transfection, PEI protects the target gene from intracellular nuclease degradation, while promoting its successful entry into the nucleus for transcription. After the successful expression of VEGF and Ang‐1 genes, they promote spontaneous colonization in HUVECs or are secreted by the transfected cells to induce proliferation and migration processes in the surrounding cells. Notably, the expression levels of VEGF and Ang‐1 were always significantly lower in the DGADSs‐2 group than in the DGADSs‐1 group (*P* < 0.05). We speculated that this might be due to the fragment size of the VEGF/Ang‐1 chimeric plasmid was much larger than that of VEGF and Ang‐1 plasmids, resulting in the ineffective translation of the VEGF/Ang‐1 chimeric plasmid.

### 3.7 Expression of angiogenesis‐related proteins in HUVECs cultured with DGADSs

The Angiogenesis Antibody Array was used to detect the expression levels of angiogenesis‐related proteins in the DGADSs‐1, DGADSs‐2, and control groups after 5 days of culture. Figure 3f–h showed that on day 5, the expression level of bFGF was significantly increased in the DGADSs‐1 and DGADSs‐2 groups; the increase was greatest in the DGADSs‐2 group. The expression levels of MMP‐1, MMP‐9, TIE2, VEGF, VEGF‐D, VEGFR‐2, and VEGFR‐3 were significantly higher in the DGADSs‐1 and DGADSs‐2 groups than in the control group; the increases were greatest in the DGADSs‐1 group. These changes in angiogenesis‐related protein expression offer insights for subsequent exploration of the corresponding signaling pathways.

### 3.8 Selection of optimal gene loading of GASs

To clarify the optimal gene loading of GASs, we used a rat subcutaneous implantation model to implant three groups of successfully constructed GASs (DGADSs‐1, DGADSs‐2, and PEI/pVEGF) into subcutaneous tissue. Through blood perfusion, gross observation, histopathology, and immunofluorescence analysis, we determined that the optimal gene loading of GASs in vivo was 10 μg/unit scaffold (*d* = 1.2 cm), which provided a basis for subsequent studies of skin defect repair (see details in the Supporting Information).

### 3.9 Effects of DGADSs on wound healing in full‐thickness skin defects

#### 3.9.1 One‐step transplantation and macroscopic observations of wounds

**FIGURE 4:**
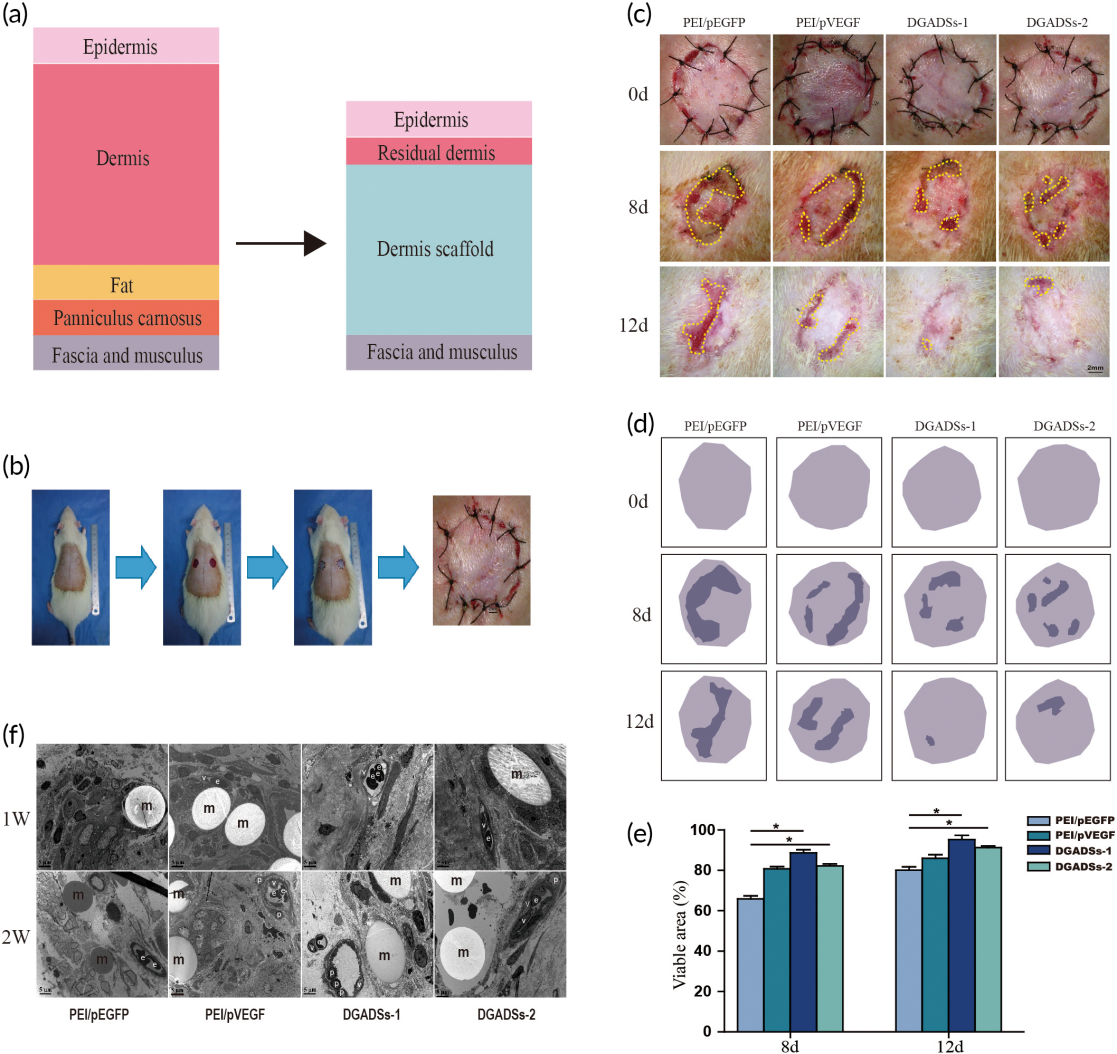
Schematic diagram, surgical operation, and gross views of one‐step transplantation model of rat full‐thickness skin defect and observation of microstructure of vessels in scaffolds: (a) schematic diagram of one‐step transplantation; (b) the surgical operation of one‐step transplantation model; (c) the gross views of the four GASs at different times after one‐step transplantation, and the yellow frame indicates the non‐viable site; (d) graphical representation of the non‐viable area of the split‐thickness skin in four GASs on postoperative days 8 and 12; (e) the quantification of the viable area of the split‐thickness skin in four GASs on postoperative days 8 and 12; (f) are TEM observation of four group scaffolds at different time points (1w and 2w) after transplantation. “m” is the cross‐sectional view of PLGA‐knitted mesh in scaffolds.

On the basis of previous studies, we loaded 10 μg of pDNA into PLGAm/CCSs via PEI, constructed four groups of GASs (PEI/pEGFP, PEI/pVEGF, DGADSs‐1, and DGADSs‐2), and transplanted them into full‐thickness skin defects on Sprague–Dawley rat dorsa. Combined with the one‐step transplantation of autologous STS, we observed their effects on promoting angiogenesis and wound healing. Figure 4a,b showed the schematic diagram and the application of one‐step transplantation into a rat full‐thickness skin defect, respectively. As shown in Figure 4c, the wound maintained a circular structure over time, but its size decreased. In the later stages, after suture removal, the shape of the wound gradually became longer and narrower; this might have been caused by excessive pressurization of gauze dressings. On the day after transplantation, the wounds in all four groups were adequately sutured without significant bleeding; the wound areas were similar among groups. On day 8, the transplanted STS in each group gradually became red and soft, without bleeding or infection. The surviving STS areas were much larger in the DGADSs‐1 and DGADSs‐2 groups than in the PEI/pVEGF and PEI/pEGFP groups. On day 12, the transplanted STS in the DGADSs‐1 and DGADSs‐2 groups survived; the surviving area was largest in the DGADSs‐1 group, followed by the DGADSs‐2 group (Figure 4d,e). The transplanted STS in the PEI/pVEGF group also survived because the pVEGF was functional. Surprisingly, the survival area in the PEI/pEGFP group was also >50%, mainly because of the synergistic effect of the mechanical support and the 3D porous structure of the PLGA/CCSs, which could rapidly induce tissue and blood vessel ingrowth.^30^

#### 3.9.2 TEM observation

At 1 and 2 weeks post‐transplantation, we observed the microstructure of vessels inside the scaffolds using TEM (Figure 4f). At 1 week post‐transplantation, we observed new vessels in the PEI/pVEGF, DGADSs‐1, and DGADSs‐2 scaffolds; these vessels were surrounded by vascular endothelial cells with small lumens and no pericytes. At that time, it was difficult to find new vessels in the PEI/pEGFP group. After 2 weeks, vessels were present in each group of scaffolds. Furthermore, pericytes appeared in the vessel walls of the PEI/pDNA‐VEGF, DGADSs‐1, and DGADSs‐2 groups, which indicated that the vessels in these three groups had changed from nascent to mature. In the PEI/pEGFP group, no pericytes were observed in the vessel walls, and the vessels were in the nascent stage.

#### 3.9.3 H&E staining, Collagen deposition and Angiogenesis in early stage of wound healing

**FIGURE 5:**
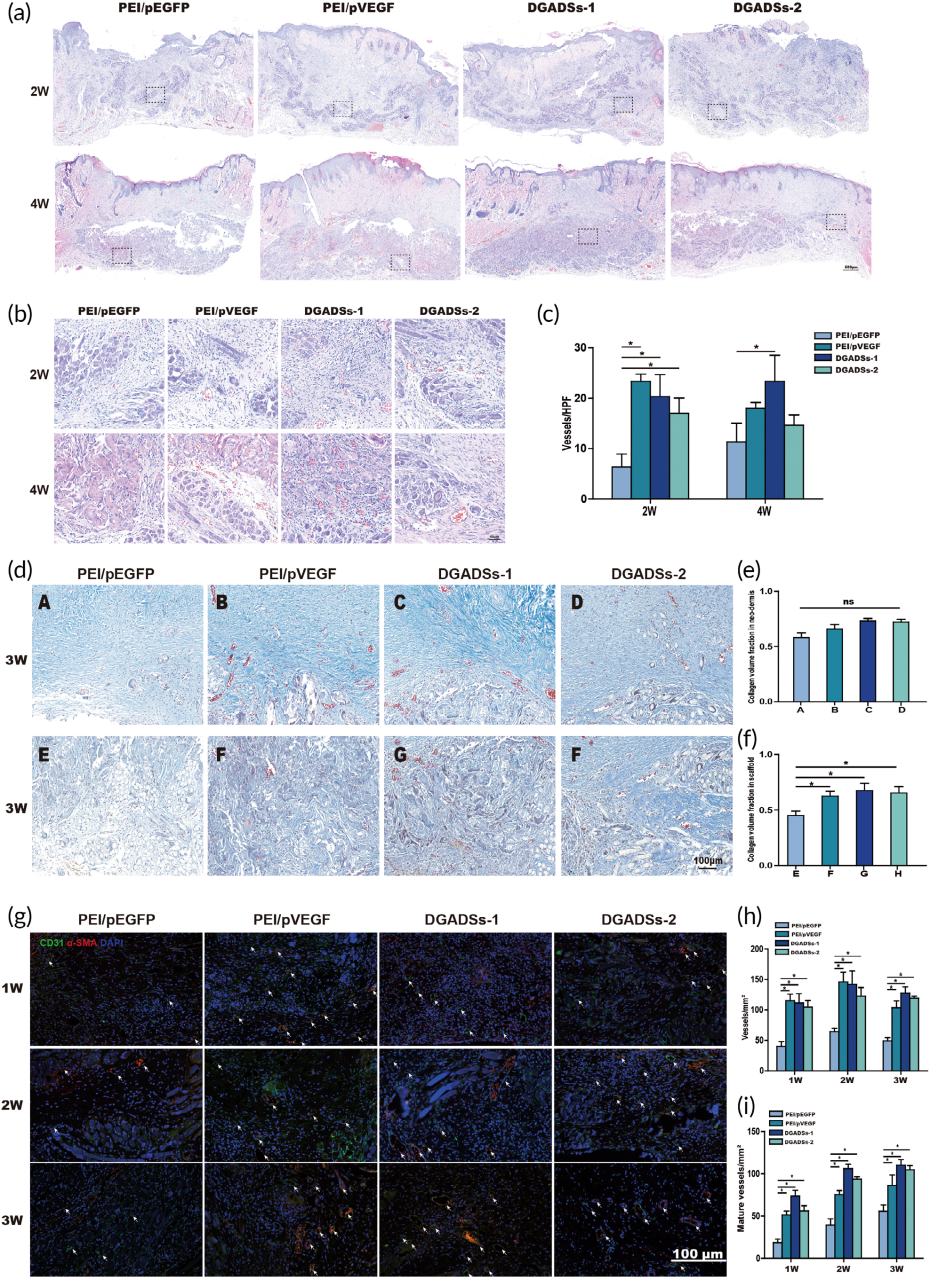
H&E staining, collagen deposition, and angiogenesis in early stage of wound healing. (a) The H&E staining results of four group scaffolds (PEI/pEGFP, PEI/pVEGF, DGADSs‐1, and DGADSs‐2) at different time points (2w and 4w) after transplantation, Scale bar = 500 μm; (b) enlarged view of the black frame within a, Scale bar = 50 μm; (c) the quantification of the vessels of four group scaffolds at different time points (2w and 4w), respectively. *p < 0.05, *n* = 4. (d) The Masson staining results of four group scaffolds (PEI/pEGFP, PEI/pVEGF, DGADSs‐1, and DGADSs‐2) at 3 weeks after transplantation. (a–d) Masson staining of new dermal tissues from different scaffolds, and (e–h) Masson staining of tissues inside different scaffolds, Scale bar = 100 μm. (e,f) Collagen quantitative analysis, respectively. *p < 0.05, *n* = 4. (g) The immunofluorescence staining results of CD31 and a‐SMA in four group scaffolds (PEI/pEGFP, PEI/pVEGF, DGADSs‐1, and DGADSs‐2) at different time points (1w, 2w, and 3w) after transplantation. CD31, a‐SMA, and cell nucleus are stained with green, red, and blue, respectively. White arrows indicate mature blood vessels. Scale bar = 100 μm. (h,i) The quantification of the total blood vessels and mature blood vessels of four group scaffolds at different time points (1w, 2w, and 3w), respectively. *P < 0.05, *n* = 4.

H&E staining of the four GAS groups at 2 and 4 weeks post‐transplantation revealed cell infiltration, scaffold residue, and angiogenesis (Figure 5a,b). PLGAm/CCSs were clearly observed in the four GAS groups at 2 weeks post‐transplantation. There were no significant differences in cell infiltration among the four GAS groups. However, the numbers of vessels were much larger in the PEI/pVEGF, DGADSs‐1, and DGADSs‐2 groups than in the PEI/pEGFP group; the number was greatest in the PEI/pVEGF group, followed by the DGADSs‐1 and DGADSs‐2 groups (Figure 5c). At 4 weeks post‐transplantation, the scaffold structure was looser in each group, but the PLGAm structure remained clearly visible. At that time, the scaffolds in each group were mainly infiltrated by fibroblasts, and the number of vessels in each group was lower than at 2 weeks post‐transplantation. Notably, the number of vessels was greatest in the DGADSs‐1 group, followed by the PEI/pVEGF, DGADSs‐2, and PEI/pEGFP groups (Figure 5c).

Figure 5d–f shows collagen deposition in the four GAS groups. Figure 5d shows Masson staining of the nascent dermis and the inside of the scaffolds in the four GAS groups at 3 weeks post‐transplantation. There was no obvious difference in collagen morphology or content among the four groups in nascent dermis (Figure 5e), but the blue collagen was darker in the DGADSs‐1 group than in the other three groups. Masson staining inside the scaffolds revealed that collagen deposition in the four groups was distributed along the pore structure of the scaffolds. The collagen contents were significantly greater in the PEI/pVEGF, DGADSs‐1, and DGADSs‐2 groups than in the PEI/pEGFP group (Figure 5f).

To evaluate the angiogenesis and maturation effects of the four groups of scaffolds, we performed CD31 and α‐SMA double‐fluorescence staining and stained‐vessel counts. CD31 is a marker of endothelial cells, which allows the assessment of endothelial cell proliferation.^31^ α‐SMA is a marker of mature pericytes that surround vascular endothelial cells; it is used as a sign of vascular maturation.^32^ As shown in Figure 5g, mature vessels with double positivity for CD31 (red) and α‐SMA (green) were visible in wounds treated with PEI/pVEGF, DGADSs‐1, and DGADSs‐2 at all time points. Moreover, DGADSs‐1 developed larger diameter vessels with thicker walls at 2 and 3 weeks after transplantation. As shown in Figure 5h, the total numbers of vessels in GAS‐transplanted wounds in all four groups first increased and then decreased. The number of vessels was greatest in the PEI/pVEGF group at 1 and 2 weeks after transplantation, after which the number of vessels increased in the DGADSs‐1 group and gradually exceeded the number in the PEI/pVEGF group. The numbers of mature vessels in the four groups of GAS‐transplanted wounds increased over time. The number of mature vessels was greatest in the DGADSs‐1 group, followed by the DGADSs‐2, PEI/pVEGF, and PEI/pEGFP groups (Figure 5i).

#### 3.9.4 H&E staining and Collagen deposition in late stage of wound healing

**FIGURE 6:**
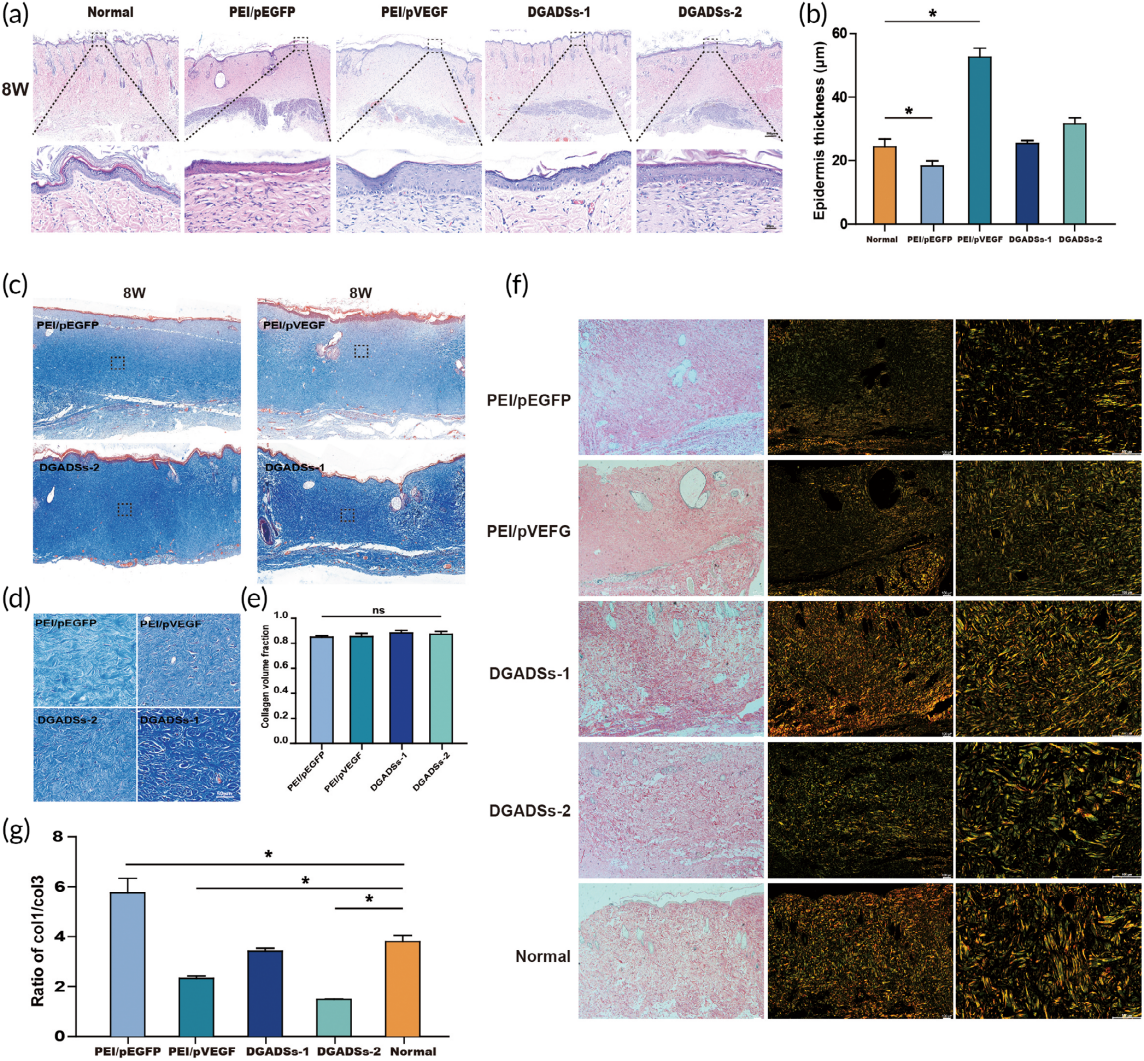
H&E staining and collagen deposition in late stage of wound healing The H&E staining results of epidermal junction of four group scaffolds at 8 weeks after transplantation, Scale bar = 500 μm. (b) The quantification of epidermal thickness. (c) The Masson staining results of four group scaffolds (PEI/pEGFP, PEI/pVEGF, DGADSs‐1, and DGADSs‐2) at 8 weeks after transplantation. (d) Enlarged view of the black frame within d, Scale bar = 50 μm. (e) Quantitative analysis of collagen, *n* = 3. (f) The Sirius Red staining results of four group scaffolds (PEI/pEGFP, PEI/pVEGF, DGADSs‐1, and DGADSs‐2) at 8 weeks after transplantation, from left to right, respectively, white light field, ×100 and ×200 magnification. (G) is the quantification of ratio of COL 1/COL 3. *P < 0.05, *n* = 3.

At 8 weeks post‐transplantation, we observed the dermal–epidermal junction and epidermal thickness in the four GAS groups. As shown in Figure 6a, the interface of the dermal–epidermal junction was flat in the PEI/pVEGF, DGADSs‐1, and DGADSs‐2 groups; additionally, new capillaries grew below the epidermis, epidermal basal cells proliferated, and the epidermis showed greater than normal thickening. The observed increase in thickness was greatest in the PEI/pVEGF group, while the epidermal thicknesses in the DGADSs‐1 and DGADSs‐ 2 groups were minimally different from the thickness of normal skin (Figure 6b). Figure 6c,d show Masson staining results in the four GAS groups at 8 weeks post‐transplantation. We found that there was no significant difference in the amount of collagen among the four groups (Figure 6e), but the blue collagen in the DGADSs‐1 group was darker than in the other three groups, and the collagen arrangement was similar to normal skin. Figure 6f shows Sirius red staining at 8 weeks after transplantation; Type 1 collagen (COL 1) and type 3 collagen (COL 3) are stained yellow and green, respectively. The COL 1/COL 3 ratio in the DGADSs‐1 group was close to the ratio in normal skin, and COL 3 expression in the PEI/pVEGF group was lower than in the other groups (Figure 6g).

#### 3.9.5 Expression of angiogenesis‐related proteins

**FIGURE 7:**
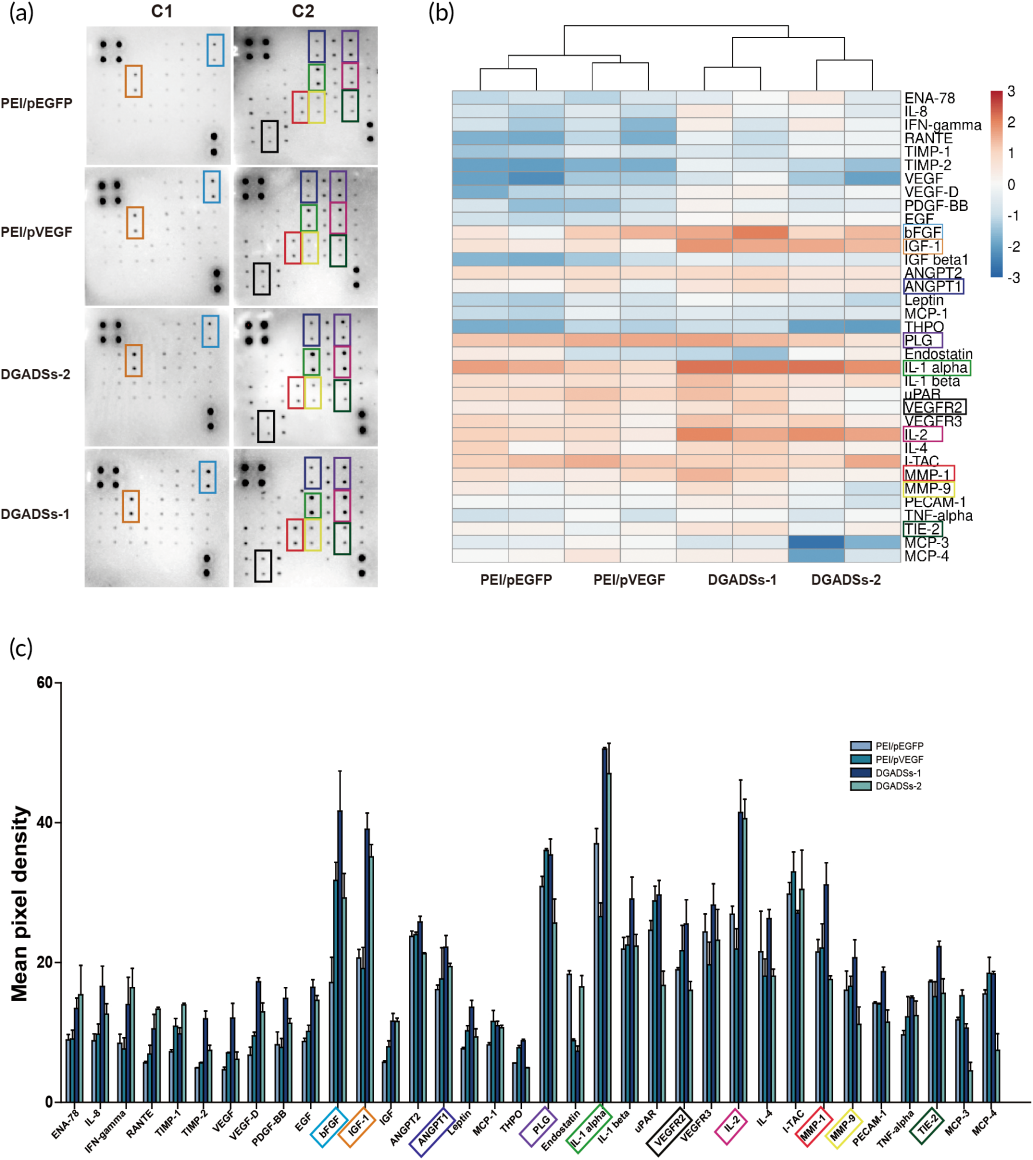
The expression of angiogenesis‐related protein in four GASs on postoperative days 14. (a) Proteome array blot of the expression of angiogenesis‐related protein in four groups of GASs lysates at initial analysis. Spot data on C1 and C2 membranes from arrays of angiogenic antibodies in PEI/pEGFP, PEI/pVEGF, DGADSs‐2, and DGADSs‐1 (From Top‐to‐bottom). (b) Heatmap recapitulating the differentially expressed in angiogenic protein between four groups of GASs. Red represents relatively high expression, and blue represents relatively low expression. Proteins of interest are highlighted in colored outlines. (c) The relative performance in angiogenic protein expression between four groups of GASs was calculated.

As shown in Figure 7a–c, the expression level of bFGF was significantly greater in the PEI/pVEGF, DGADSs‐1, and DGADSs‐2 groups than in the PEI/pEGFP group at 2 weeks post‐transplantation; the increase was greatest in the DGADSs‐1 group. The expression levels of IGF‐1, PDGF‐BB, VEGF‐D, EGF, ANGPT‐1, IL‐1a, and IL‐2 were significantly higher in the DGADSs‐1 and DGADSs‐2 groups than in the other two groups; these increases were also greatest in the DGADSs‐1 group. In addition, the expression levels of MMP‐1, MMP‐9, TIE2, VEGF, and VEGFR‐2 were higher in the DGADSs‐1 group than in the other three groups.

## 4 DISCUSSION

Through general observation, histopathology, immunofluorescence, and other detection methods, we studied the effects of GASs with different pDNA gene loading levels on angiogenesis, maturation, and wound healing (details in the Appended Material section). Our results showed that the unit scaffold (*d* = 1.2 cm) with gene loading in the range of 5–15 μg promoted cell proliferation, collagen deposition, and angiogenesis; among the tested concentrations, the effect of 10 μg pDNA was greatest. Notably, the pDNA‐15‐μg scaffold was less effective than the pDNA‐10‐μg scaffold in promoting tissue repair and vascularization. The VEGF dose is strictly regulated during vascular development.^33^ Overexpression of VEGF by various gene therapy vectors reportedly results in the growth of aberrant angioma‐like vascular structures in various tissues, including skeletal muscle^34^, ^35^ and the myocardium.^34^, ^36^ Preclinical data have also indicated a surprisingly narrow therapeutic window for VEGF gene delivery, such that low vector doses are safe but not sufficiently effective to yield a therapeutic benefit, whereas slightly higher doses rapidly become unsafe.^34^ Subsequent studies have revealed that the spatial distribution of VEGF in tissue is critical for determining its therapeutic window.^37^, ^38^ Indeed, the switch between normal and aberrant angiogenesis by VEGF, as well as its therapeutic potential, depends strictly on the amount of factor localized in the microenvironment around each producing cell, rather than the total dose. This is because VEGF binds tightly to the extracellular matrix,^39^ such that heterogeneous levels are unbalanced in tissue. Therefore, “hot spots” of excessive expression can have toxic effects, despite low total doses of VEGF. Other studies have confirmed that growth factors show dose‐dependent effects on the growth of cells and tissues. For example, human fibroblast growth factor‐2 (80 ng) has a two‐dimensional spatiotemporal regulatory effect on the growth of blood and lymphatic capillaries during corneal tissue reconstruction in mice; when the dose was reduced to 12.5 ng, lymphangiogenesis was predominant, which is related to VEGF‐C and VEGF‐D.^40^ Therefore, in this study, we selected the 10 μg/unit scaffold for the follow‐up experiment.

Our in vivo results indicated that various PEI/pDNA NPs should be used in combination with a delivery carrier. Compared with pVEGF alone or the chimeric plasmid pVEGF/Ang‐1, DGADSs loaded with pVEGF and pAng‐1 induced greater vessel formation. DGADSs‐1 can sustainably release pVEGF and pAng‐1 to consistently transfect cells surrounding the wound; they also facilitate angiogenesis, vascular maturation, and tissue repair.^41^, ^42^ Therefore, a higher level of vascularization can be realized to achieve a more desirable wound repair effect when both VEGF and Ang‐1 are present than when only one is present. Notably, DGADSs‐2 loaded with VEGF/Ang‐1 chimeric plasmid exhibited a vascularization effect distinct from the effect of DGADSs‐1. A possible explanation for this finding involves the induction of vascularization after inflammation.^8^ In this context, although VEGF/Ang‐1 chimeric plasmid retains the angiogenic characteristics of VEGF, it induces significantly fewer angioma‐like structures, with less inflammation.^13^ An alternative explanation for the above finding is that VEGF can bind specifically to VEGFR‐2 receptors on the surface of endothelial cells to promote endothelial cell migration and proliferation, followed by primary lumen formation.^43^ Ang‐1 initiates positive regulation of vascular differentiation and maturation through specific binding to Tie‐2 receptors.^13^ Studies have shown that VEGF/Ang‐1 binds VEGFR‐2, Tie‐2, and VEGFR‐1. The affinity of VEGF/Ang‐1 for VEGFR‐2 is comparable to the affinity of VEGF for VEGFR‐2^44^; the affinity of VEGF/Ang‐1 for Tie2 is similar to the affinity of the Ang‐1 fibrinogen‐like domain for Tie‐2.^45^ Therefore, VEGF/Ang‐1 is involved in competitive binding. The above experimental results demonstrated that the effects of promoting vascularization and tissue repair were similar in the DGADSs‐2 and PEI/pVEGF groups; thus, we speculate that VEGF/Ang‐1 combines mainly with VEGFR‐2, and partially with Tie‐2.

The Angiogenesis Antibody Array analysis showed that the expression levels of bFGF, IGF‐1, PDGF‐BB, VEGF‐D, EGF, ANGPT‐1, IL‐1a, IL‐2, MMP‐1, MMP‐9, TIE2, VEGF, and VEGFR‐2 were increased; the relative expression level was highest in the DGADSs‐1 group. These results were consistent with the cell experiments in vitro, H&E, and fluorescence staining findings described above, indicating positive associations of these factors with VEGF and Ang‐1 angiogenesis. bFGF could stimulate the proliferation of fibroblasts and vascular endothelial cells, thus promoting angiogenesis and wound healing. Angiogenesis in wounds can be completely blocked when bFGF is inhibited^46^; IGF‐1 reportedly has a central role in the expression of VEGF. In addition, IGF‐l can upregulate the expression and protein secretion of VEGF by upregulating the transcription of VEGF mRNA.^47^ MMP‐9 is mainly secreted by fibroblasts and inflammatory cells; it can degrade basement membrane and extracellular matrix, which are involved in angiogenesis. In angiogenesis, MMP‐9 may promote the migration of vascular endothelial cells by degrading the vascular basement membrane and extracellular matrix. VEGF and MMP‐9 have demonstrated important synergistic effects on angiogenesis. MMP‐9 can promote the upregulation of VEGF expression in endothelial cells and cause VEGF to more strongly act on its receptors, thereby inducing angiogenesis; however, the underlying mechanism requires further investigation.^48^

Moreover, analysis of the results showed that the expressions of related inflammatory factors (TNF‐α and IL‐6) in the GASs group were higher than those in the blank control group. Inflammation is an important stage in wound healing. At the early stage of inflammation, neutrophils infiltrate, and then, monocyte/macrophage system is activated and recruited to the wound.^49^ Macrophages are the cellular hubs of inflammation and angiogenesis. In the early stage of wound healing, macrophages secrete inflammatory factors, such as TNF‐α and IL‐6, to mediate the wound inflammation. IL‐6 and TNF‐α play a central role in inflammation by nuclear factor‐kappa B (NF‐κB) signal transduction and transcriptional activator factor 3 (STAT3). Studies have found that inflammation and angiogenesis have many common signaling pathways. Such as the IL‐6‐STAT3 pathway, which was involved in the epithelial‐mesenchymal transition (EMT),^50^ and the induction of hypoxia‐inducible factor‐1α (HIF‐1α) and VEGF, two key players for angiogenesis.^51^ Chen et al. indicated that ADSC and its secretory factor IL‐6 were effective for enhancing skin flap recovery and angiogenesis after ischemia/reperfusion injury.^52^ COX/prostaglandin pathway, prostanoids generated by COX‐2 can induce the expression of VEGF and other pro‐angiogenic factors.^53^ In conclusion, inflammatory factors may directly induce vessel formation via engagement of target endothelial cells or, indirectly, by inducing leukocytes and/or endothelial cells to produce proangiogenic mediators. Thus, one can say angiogenesis and inflammation are interlinked.

In summary, the mechanisms of DGADSs in promoting angiogenesis, vessels maturation, and wound healing are complex, involving inflammatory reaction, numerous cells, growth factors and cytokines; they require further study.

## 5 CONCLUSIONS

By loading PEI/pDNA NPs on PLGAm/CCSs, we successfully constructed two types of DGADSs. Our in vitro analyses showed that both DGADSs had good biocompatibility and could continuously release PEI/pDNA nanoparticles; they demonstrated effective transfection ability and could upregulate the expression levels of VEGF and Ang‐1. Our in vivo experiments showed that DGADSs loaded with pVEGF and pAng‐1 promoted angiogenesis and wound healing more efficiently than did DGADSs loaded with pVEGF alone or with the pVEGF/Ang‐1 chimeric plasmid alone. The Angiogenesis Antibody Array analysis showed that the angiogenic and tissue repair effects of DGADSs might be related to increases in the expression levels of bFGF, VEGF, VEGF‐D, TIE2, and VEGFR‐2, thus providing insights for the subsequent exploration of the corresponding signaling pathways. Therefore, DGADSs may enable the vascularization of engineered tissue and promote the development of wound repair substitutes.

## AUTHOR CONTRIBUTIONS

**Tingting Weng:** Data curation (lead); methodology (lead); writing – original draft (lead); writing – review and editing (lead). **Min Yang:** Data curation (lead); methodology (lead); writing – original draft (supporting); writing – review and editing (supporting). **Wei Zhang:** Data curation (equal); formal analysis (equal); methodology (supporting); writing – review and editing (supporting). **Ronghua Jin:** Formal analysis (equal); methodology (supporting); writing – review and editing (supporting). **Sizhan Xia:** Methodology (supporting); software (supporting); writing – review and editing (supporting). **Manjia Zhang:** Methodology (supporting); writing – review and editing (supporting). **Pan Wu:** Funding acquisition (supporting); writing – review and editing (supporting). **Xiaojie He:** Funding acquisition (supporting). **Chunmao Han:** Funding acquisition (supporting); writing – review and editing (supporting). **Xiong Zhao:** Writing – review and editing (supporting). **Xingang Wang:** Data curation (lead); methodology (lead); writing‐review and editing (lead); funding acquisition (supporting).

## CONFLICT OF INTEREST STATEMENT

The authors declare no conflict of interest.

### PEER REVIEW

The peer review history for this article is available at https://www.webofscience.com/api/gateway/wos/peer-review/10.1002/btm2.10562.

## Supporting information


**Data S1.** Supporting Information.

## Data Availability

The authors declare that all data supporting this work is available in the paper and supporting information.
