# Supplementary material for: Dual gene‐activated dermal scaffolds regulate angiogenesis and wound healing by mediating the coexpression of VEGF and angiopoietin‐1
Source: Bioeng Transl Med. 2023 Jun 25;8(5):e10562. doi: 10.1002/btm2.10562 (PMC10487340; doi:10.1002/btm2.10562)
Supplement: Supplementary file 1 — Data S1. Supporting Information. [file BTM2-8-e10562-s001.docx]

**Dual gene-activated dermal scaffolds regulate angiogenesis and wound healing by mediating the coexpression of VEGF and angiopoietin-1**

Tingting Weng ^1, 2†^ , Min Yang^1, 2†^, Wei Zhang^1, 2^, Ronghua Jin^1, 2^, Sizhan Xia^1, 2^, Manjia Zhang^3^, Pan Wu^1, 2,^, Xiaojie He^1, 2^, Chunmao Han^1, 2,^, Xiong Zhao^4^, Xingang Wang^1, 2,*^

^1^ Department of Burns & Wound Care Centre, Second Affiliated Hospital of Zhejiang University School of Medicine, Hangzhou 310009, China

^2^ The Key Laboratory of Severe Trauma and Burns of Zhejiang Province, 310009, China

^3^ The First Clinical Medical College, Zhejiang Chinese Medical University, Hangzhou 310053, China

^4^ Department of Burn and Plastic Surgery, Children's Hospital, Zhejiang University School of Medicine, National Clinical Research Center for Child Health, National Children's Regional Medical Center, Hangzhou 310052, China

* Corresponding author. Address: Department of Burns & Wound Care Center, Second Affiliated Hospital of of Zhejiang University, College of Medicine, Hangzhou, China, 310009.

† These authors contributed equally to this work

Tel: +86 571 87767187; Fax: +86 571 87784585;

E-mail: [wangxingang8157@zju.edu.cn](mailto:wangxingang8157@zju.edu.cn)

**Figure S1** showed the schematic diagram of plasmid map.

Through subcutaneous implantation of GASs in rats, the influence of GASs on vascularization of dermal scaffold in vivo was evaluated, and the appropriate gene loadings per scaffold was determined. On the 10 and 21 days after implantation, blood flow signals of subcutaneous implanted scaffolds were detected by small animal ultrasound Doppler (**Figure S2**). The results showed that the blood flow signals on the scaffolds surface of pDNA-10ug and pDNA-15ug groups were significantly stronger than those of pDNA-5ug and pDNA-0ug groups on the day 10. On the day 21 after implantation, the blood flow signals of the four groups of stents all decreased. Then, the blood flow signal of pDNA-10ug was still higher than that of the other three groups.

**Figure S3** showed the gross views of the 3 groups of GASs implanted 10 and 21 days. In general, the scaffolds wrapped in subcutaneous tissue basically maintains a circular structure, and the PGAm mesh can be observed, and new blood vessels were attached to the surface of the scaffolds. On the day 10 after implantation, the diameter of the scaffolds /tissue wrap in each group were maintained at approximately 10.3±0.7cm, and the boundary with surrounding tissues was clear, the shape remained basically unchanged, and the surface blood vessels increased. The two groups of pDNA-5ug and pDNA-10ug had relatively more blood vessels on the surface of the scaffolds, and the diameter of the blood vessels was also thicker. The pDNA-0ug and pDNA-15ug groups had relatively few blood vessels on the surface of the scaffolds. On the day 21 after implantation, the diameter of the scaffolds/tissue wrap were about 8.3±0.5cm in each group, making the boundary with the surrounding tissue more blurred. The vessels on the scaffolds surface significantly resolved in the four groups. However, there were relatively more blood vessels on the scaffolds surface in the pDNA-10ug group compared with the other 3 groups. The above results can roughly confirm that the pDNA-10ug group has the best angiogenesis effect.

Wound samples were collected on postoperative days 14, and 21 for histological analysis to investigate the effect of GASs loaded with different amounts of pDNA on wound healing (**Figure S4 and S5**). A small number of fibroblasts and collagen were deposited on pDNA-0ug on the 14 days after implantation. The infiltration of Fbs was significant and collagen deposition increased in the other three groups. The surrounding normal tissues rapidly developed to the center of the scaffold. And pDNA-10ug was the most significant(p<0.01), followed by pDNA-15ug, and then pDNA-5ug. On the 21 days after implantation, the number of infiltrated fibroblasts in the scaffold continued to increase and the collagen deposition was more significant in each group, especially in the pDNA-10ug scaffold (p<0.01). The above results suggested that pDNA-10ug had better effects on promoting cell infiltration into the scaffold and collagen deposition.

In order to evaluate the pro-vascularization effect of the VEGF gene-activated scaffolds and the optimal dose range more comprehensively, we used CD31 and a-SMA fluorescence double staining to observe the number of new blood vessels (**Figure S6**). At day 14, the vessel density of the pDNA-10ug group was found to be the highest, but had no significant difference with that of the pDNA-15ug group. They are both significantly larger than that of the pDNA-0ug and pDNA-5ug. At day 21, the vessel densities of the pDNA-10ug group were significantly higher than those of other groups of the same time. At all the time intervals, the significant difference (p<0.01) was found between the pDNA-0ug and pDNA-5ug groups in terms of the vessel density, implying that incorporation of the pDNA had effect on generation of vessels in the scaffolds. It had to mention that the vessel density in the three groups of scaffolds loaded with pDNA did not increase unlimitedly. With the prolongation of implantation time, the vessel density of scaffolds in each group decreased gradually (Figure S5B-5C). From 14 days to 21 days after implantation, the density of mature blood vessels in each group increased gradually, but their absolute values were smaller than that of the total number of vessels. Moreover, the number of mature vessels of three groups of scaffolds loaded with pDNA were always significantly higher than pDNA-0ug groups in all the detection time. Among them, the number of mature vessels loaded with pDNA-10ug was consistently significantly higher than the other groups on 21 days (p<0.05), and there were no significant differences from pDNA-15ug on 14 days.

Through general observation, histopathology, immunofluorescence and other detection methods, we studied the effects of GADSs with different pDNA gene loading on the proliferation of histiocytes, as well as angiogenesis and maturation. The results showed that the unit scaffold (d=1.2cm) with gene loading in the range of 5-15 ug had relatively obvious effects of promoting cell proliferation, collagen deposition and angiogenesis. Among them, the effect of pDNA-10ug was the most significant.

**Appendix figure**

**Figure S1**


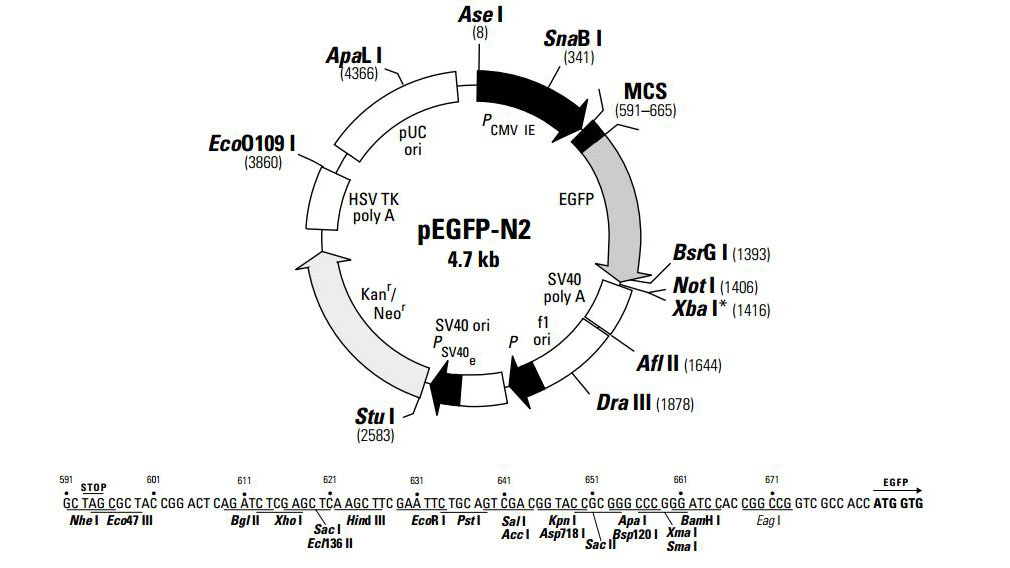


The schematic diagram of plasmid map.

**Figure S2**


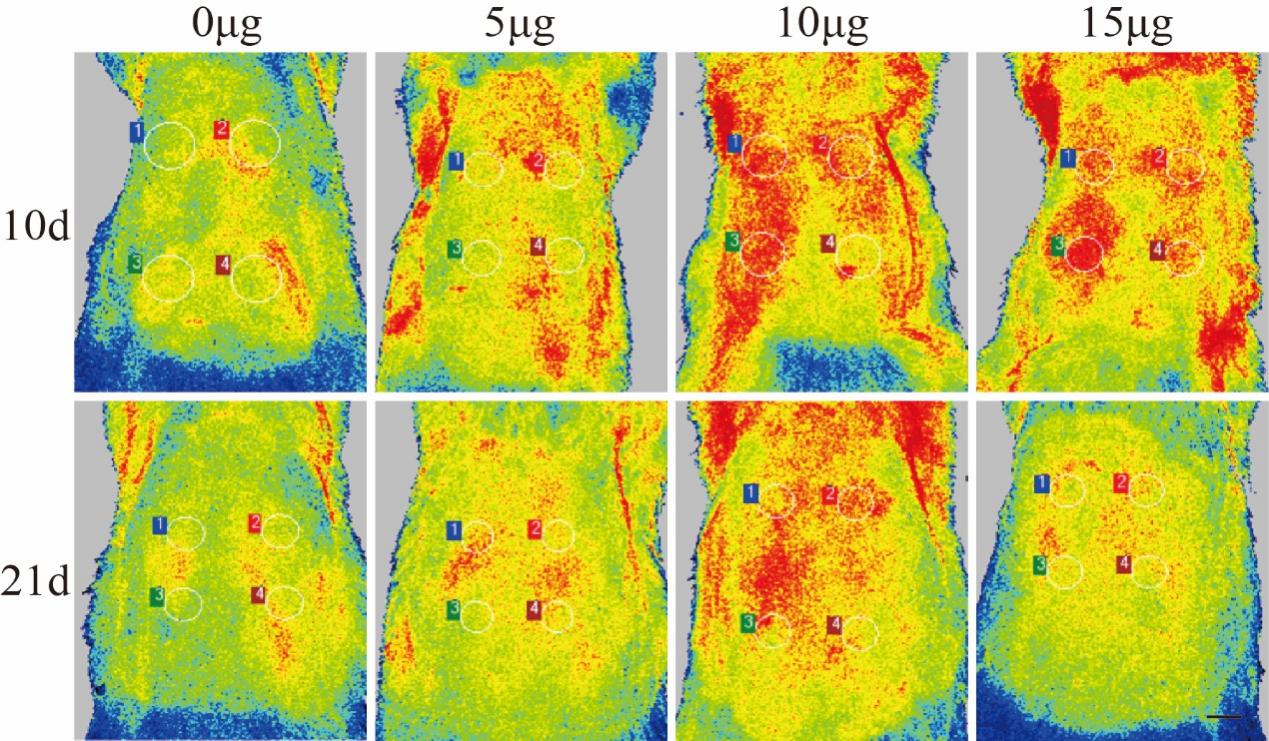
The images of ultrasound Doppler subcutaneous blood flow at 10 and 21 days for GASs with

different gene loadings (0ug, 5ug, 10ug, 15ug). Scale bar = 1cm

**Figure S3**


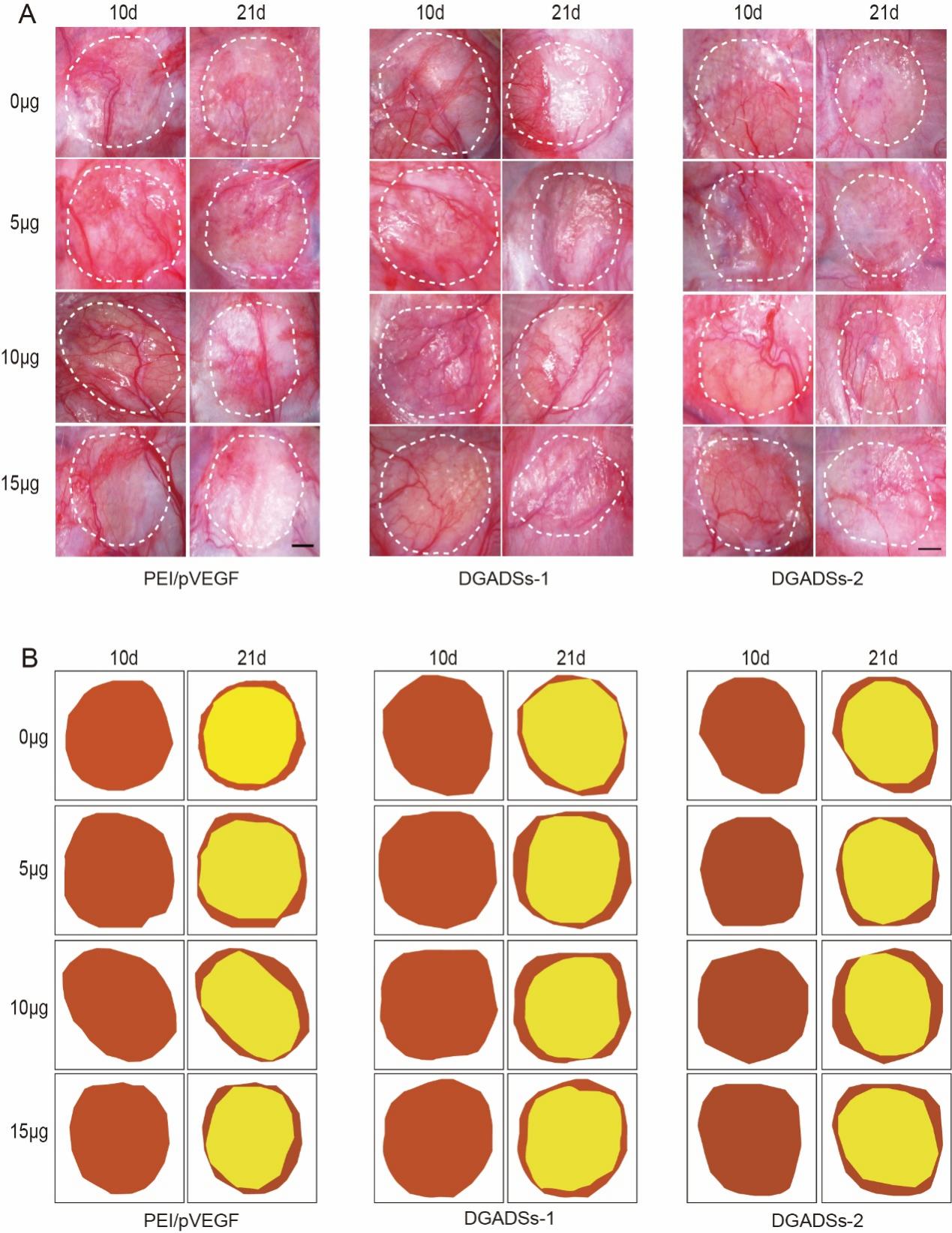


1. The gross views of the 3 groups of GASs with different gene loadings (0ug, 5ug, 10ug and

15ug) on day 10 and 21 post implantation. Scale bar = 2mm. (B) Schematic diagram of the area of the 3 groups of GASs on day 10 and 21 post implantation.

**Figure S4**


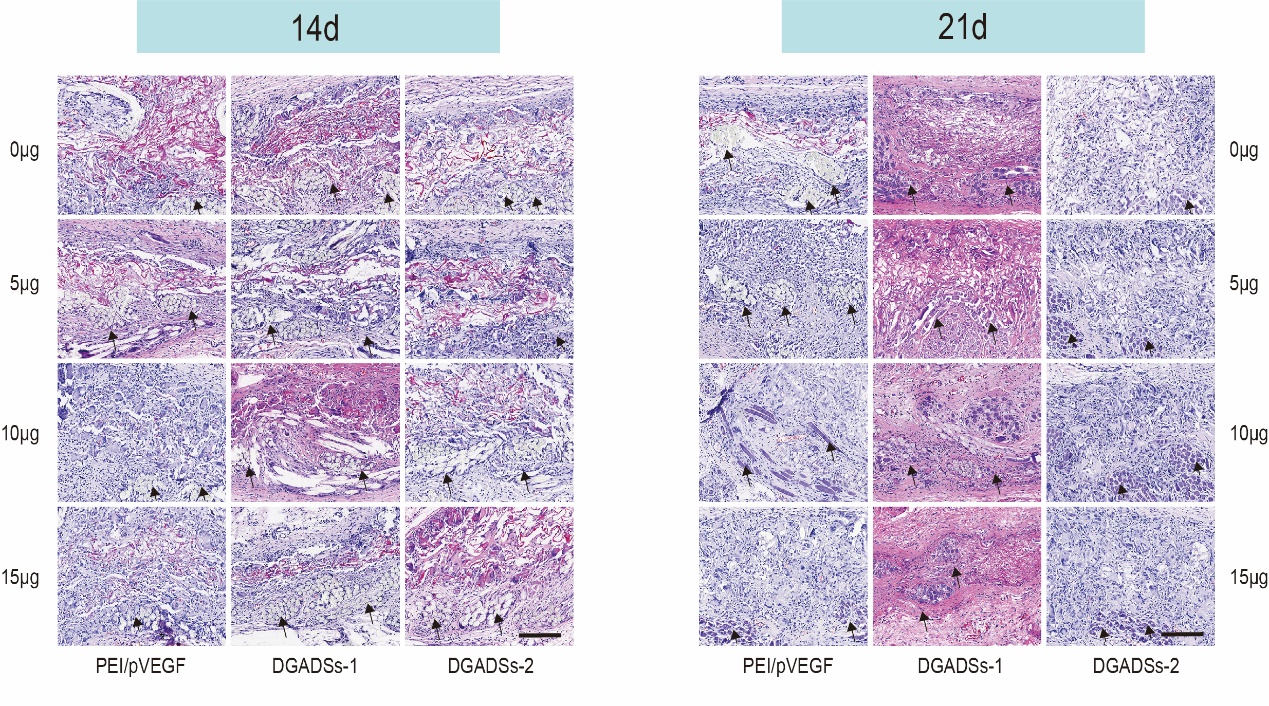


The results of HE staining of the 3 groups of GASs with different gene loadings (0ug, 5ug, 10ug and 15ug) on day 14 and 21 post implantation. Scale bar =200μm.Black arrows indicate scaffolds.

**Figure S5**


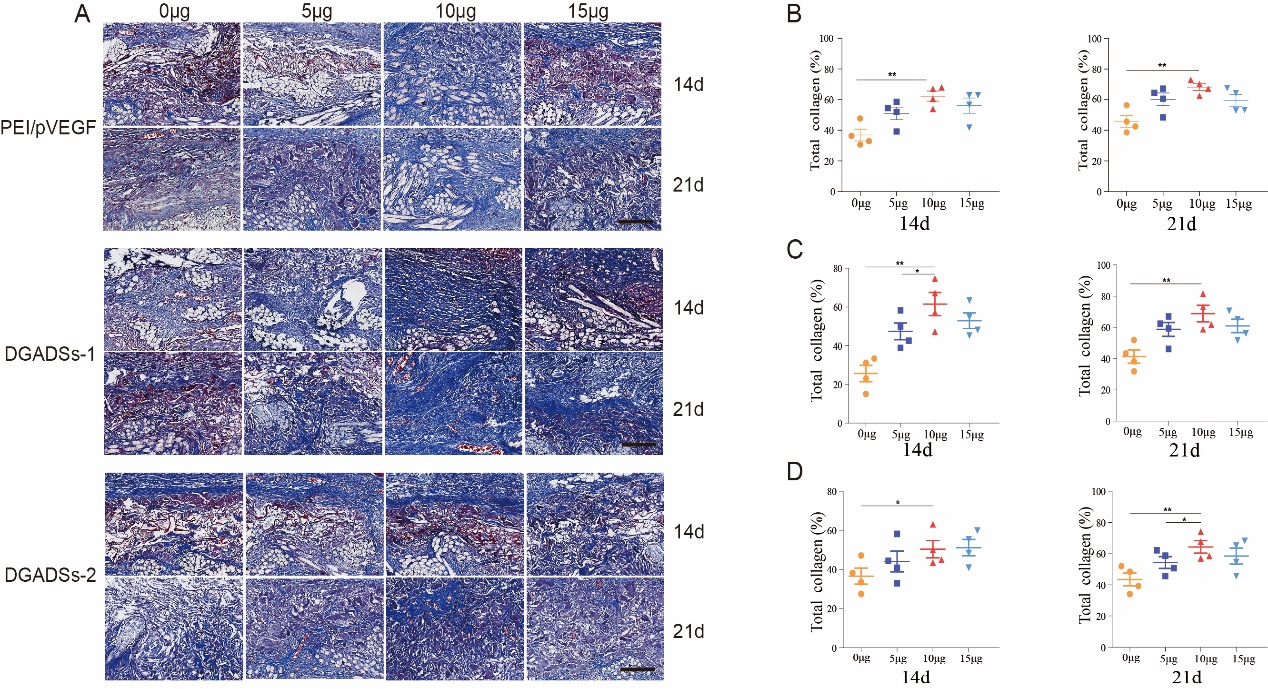


(A) The results of Masson staining of the 3 groups of GASs with different gene loadings (0ug, 5ug, 10ug and 15ug) on day 14 and 21 post implantation. Scale bar =200μm.(B-D) The quantification of total collagen of the 3 groups of GASs with different gene loadings (0ug, 5ug, 10ug and 15ug) on day 14 and 21 post implantation, respectively. * *p*<0.05 and ** *p*<0.01

**Figure S6**


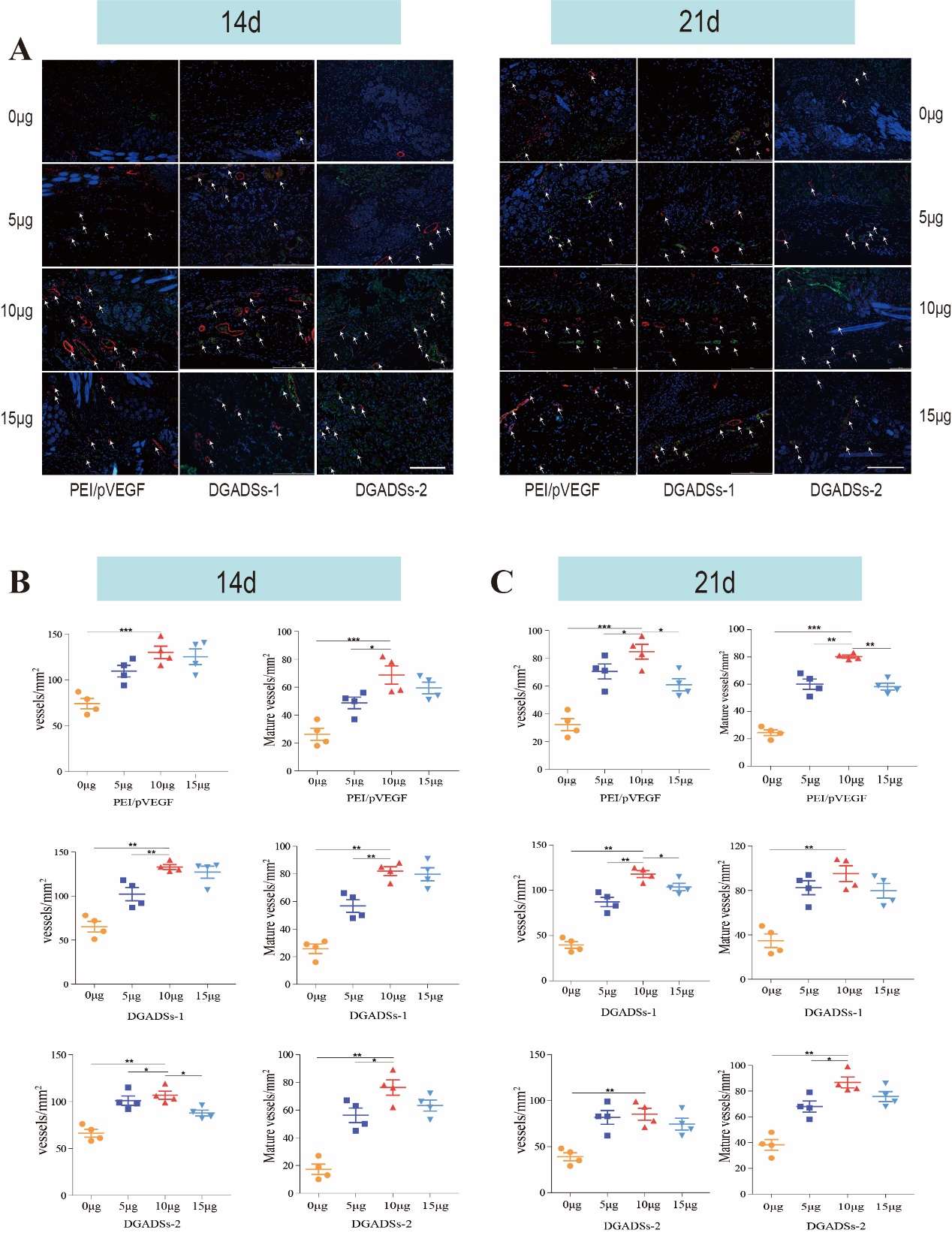


(A) The immunofluorescence staining results of the 3 groups of GASs with different gene loadings (0ug, 5ug, 10ug and 15ug) on day 14 and 21 post implantation. CD31, a-SMA and cell nucleus are stained with green, red and blue, respectively. White arrows indicate blood vessels. Scale bar = 100μm. (B-C) The quantification of the total blood vessels and mature blood vessels of the 3 groups of GASs with different gene loadings (0ug, 5ug, 10ug and 15ug) on day 14 and 21 post implantation, respectively. * *p*<0.05, ** *p*<0.01 and *** *p*<0.001.

**Figure S7**


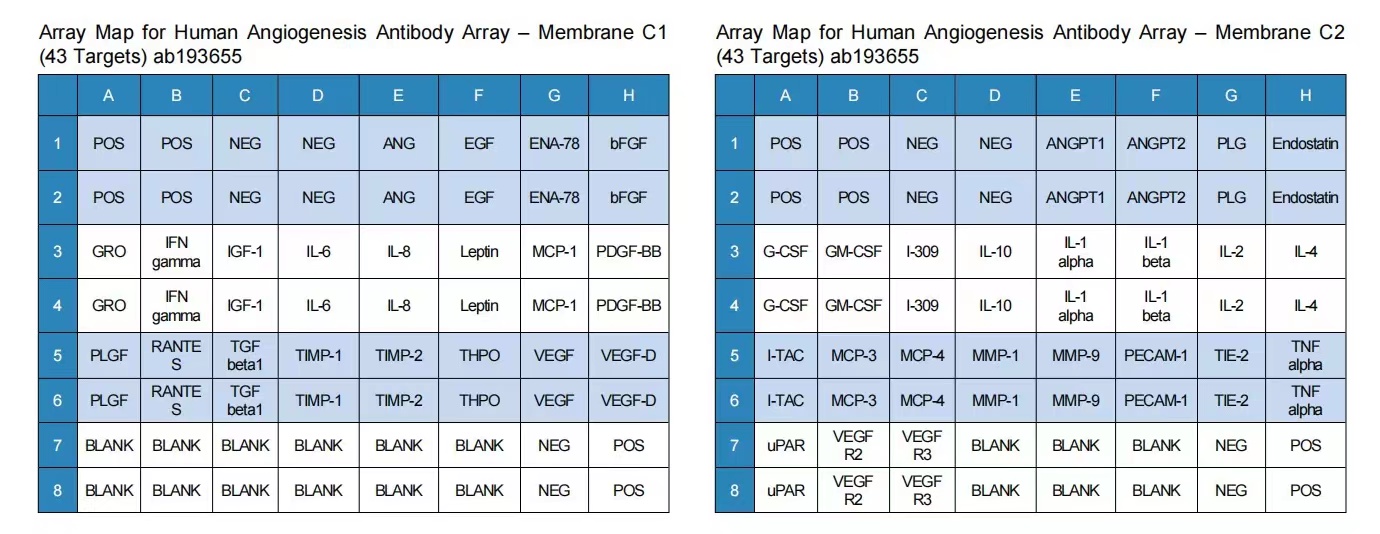


Array Map for Human Angiogenesis Antibody Array – Membrane C1 and C2 (43 Targets).

POS:Positive Control, NEG:Negative Control, BLANK:No Antibody
